# Soluble Urokinase Plasminogen Activator Receptor (suPAR) as a Biomarker of Systemic Chronic Inflammation

**DOI:** 10.3389/fimmu.2021.780641

**Published:** 2021-12-02

**Authors:** Line Jee Hartmann Rasmussen, Jens Emil Vang Petersen, Jesper Eugen-Olsen

**Affiliations:** ^1^ Department of Clinical Research, Copenhagen University Hospital Amager and Hvidovre, Hvidovre, Denmark; ^2^ Department of Psychology and Neuroscience, Duke University, Durham, NC, United States; ^3^ Division of Infectious Diseases, Duke University School of Medicine, Durham, NC, United States

**Keywords:** inflammation, biomarkers, inflammation mediators - blood, C-reactive protein, interleukin-6, inflammaging, immunosenescence

## Abstract

Systemic chronic inflammation (SCI) is persistent, health-damaging, low-grade inflammation that plays a major role in immunosenescence and in development and progression of many diseases. But currently, there are no recognized standard biomarkers to assess SCI levels alone, and SCI is typically measured by combining biomarkers of acute inflammation and infection, e.g., CRP, IL-6, and TNFα. In this review, we highlight 10 properties and characteristics that are shared by the blood protein *soluble urokinase plasminogen activator receptor* (suPAR) and SCI, supporting the argument that suPAR is a biomarker of SCI: (1) Expression and release of suPAR is upregulated by immune activation; (2) uPAR and suPAR exert pro-inflammatory functions; (3) suPAR is associated with the amount of circulating immune cells; (4) Blood suPAR levels correlate with the levels of established inflammatory biomarkers; (5) suPAR is minimally affected by acute changes and short-term influences, in contrast to many currently used markers of systemic inflammation; (6) Like SCI, suPAR is non-specifically associated with multiple diseases; (7) suPAR and SCI both predict morbidity and mortality; (8) suPAR and SCI share the same risk factors; (9) suPAR is associated with risk factors and outcomes of inflammation above and beyond other inflammatory biomarkers; (10) The suPAR level can be reduced by anti-inflammatory interventions and treatment of disease. Assessing SCI has the potential to inform risk for morbidity and mortality. Blood suPAR is a newer biomarker which may, in fact, be a biomarker of SCI since it is stably associated with inflammation and immune activation; shares the same risk factors as many age-related diseases; is both elevated by and predicts age-related diseases. There is strong evidence that suPAR is a prognostic marker of adverse events, morbidity, and mortality. It is associated with immune activity and prognosis across diverse conditions, including kidney disease, cardiovascular disease, cancer, diabetes, and inflammatory disorders. Thus, we think it likely represents a common underlying disease-process shared by many diseases; that is, SCI. We review the supporting literature and propose a research agenda that can help test the hypothesis that suPAR indexes SCI, with the potential of becoming the new gold standard for measuring SCI.

## Introduction

Systemic chronic inflammation (SCI) refers to persistent, low-grade inflammation, and it is involved in the pathogenesis of a wide variety of chronic non-communicable diseases that collectively constitute the leading cause of death globally (1). The diseases associated with SCI range from physical health problems, including cardiovascular disease, type 2 diabetes, cancer, and neurodegenerative disorders (2), to mental health disorders, such as depression, anxiety, and schizophrenia (3, 4). Assessing the level of SCI is therefore of utmost importance as it can provide information on disease burden as well as risk of incident disease, disease progression, and ultimately mortality (2).

The risk of developing SCI can be traced back to childhood development and is promoted by genetic, biological, social, environmental, and lifestyle factors (2). But even though the effects of SCI have been shown to persist throughout life with heightened risk of morbidity and mortality to follow, there are currently no recognized standard biomarkers to indicate and assess the level of SCI (2).

### Inflammation

The textbook example of inflammation is an essential immune response aimed at eliminating pathogens, clearing infections, and promoting tissue repair and recovery. This acute inflammatory response is normally a short-term process that serves to protect the host, by coordinating the delivery and activation of immune mediators (plasma proteins and leukocytes, mainly neutrophils) to the site of infection or injury. Under normal circumstances, a successful acute inflammatory response is temporally restricted and results in resolution, repair, and restoration of homeostasis once the threat has been resolved (5). If the acute inflammatory response fails to eliminate the pathogens, foreign bodies, or other causes of tissue damage (for example, persistence of self-antigens in an autoimmune response), the result is a chronic inflammatory state where active inflammation fails to resolve. The chronic inflammatory state is characterized by replacement of the neutrophil infiltrate with macrophages and lymphocytes and in some cases with formation of granulomas and tertiary lymphoid tissues (5).

In contrast to these localized inflammatory responses, *systemic* chronic inflammation is a state of persistent, low-grade immune activation affecting multiple physiological systems. While it can be caused by chronic infections, SCI can also be partly sterile, i.e., it can be triggered in the absence of infectious agents and pathogen-associated molecular patterns (PAMPs). Damage-associated molecular patterns (DAMPs) from excess glucose, cholesterol crystals, or cellular breakdown products are known to trigger the response (2, 5). SCI is characterized by chronically elevated levels of inflammatory cytokines, chemokines, and acute-phase proteins, and this persistent inflammation can end up damaging tissues and organs (2, 5). Thus, the clinical consequences of SCI are linked to a variety of disorders across organ systems and include increased risk of physical frailty, morbidity, and mortality (2).

SCI increases with age, but also chronic infections, microbiome dysbiosis, classic unhealthy lifestyle behaviors (smoking, physical inactivity, unhealthy diet), and obesity have been linked to the presence and promotion of SCI (2). Recently, social, psychological, and environmental factors including disturbed sleep, social isolation, psychological stress, and exposure to environmental or industrial toxicants (such as air pollutants) have been associated with SCI (2). Additionally, early development and childhood circumstances have been shown to promote SCI in adulthood (2). The long list of risk factors shows that there are many potential intervention targets for reducing SCI.

### (Lack of) Biomarkers for Systemic Chronic Inflammation

Despite the established link between SCI and disease, there are no standard biomarkers for SCI (2). The causes and mechanisms of SCI are poorly understood (2, 5)—in part due to the lack of precise consistent diagnostic criteria or ways to measure SCI. At the moment, the measurement of SCI for clinical or research purposes is primarily carried out by assessing biomarkers of infection or acute inflammation, mainly C-reactive protein (CRP) or fibrinogen and other acute-phase proteins, white blood cell count, erythrocyte sedimentation rate, or cytokines, such as interleukin (IL)-1β, IL-6, and tumor necrosis factor α (TNFα). The current standard for indicating presence of systemic chronic low-grade inflammation is slightly elevated CRP levels (>3 mg/L) measured with high-sensitivity CRP (hs-CRP) tests (6, 7). Alternatively, many studies use composite measures combining canonical biomarkers of acute inflammation when attempting to assess SCI (2). Both approaches have limitations. When using markers that are both sensitive to acute infection and systemic inflammation, acute influences such as undetected infections may be misinterpreted as SCI, or conversely, presence of infection may mask any underlying SCI resulting in a failing to notice actual SCI and misclassifying this as an acute infection; both situations with the result that accurate quantification of the actual level of SCI is obscured. Moreover, many of these inflammatory markers are short-lived and rapidly up- and down-regulated, as their biological function is to tightly control the acute inflammatory response. Their inherent volatility makes their quantification time-sensitive and complicates clinical interpretation. Two high-dimensional measures, the immune aging ‘IMM-AGE’ and the inflammatory aging clock ‘iAge’ based on multi-omics cellular immune profiling and deep-learning analysis of 50 inflammatory proteins, respectively, were recently shown to track SCI outcomes, but measures like these are complex and hard to apply broadly in other settings, requiring gene expression data to approximate these measures in cohorts that are less comprehensively phenotyped for immune measures (8, 9).

The lack of good stable biomarkers for SCI has the implication that there is no operational definition of SCI, and thus an individual cannot be diagnosed with *systemic chronic inflammation*. This poses a serious problem, as there is a consensus that SCI is both a major risk factor and causally involved in the pathogenesis of many diseases, in addition to being a hallmark of *immunosenescence*, the age-related decline in immune function. Ultimately, emerging pathological processes may fly under the radar and go unnoticed for too long, wasting potential windows of opportunity for treatment or interventions that could slow the course of disease—or completely prevent disease development.

But what constitutes a good biomarker of SCI? First, a biomarker of SCI needs to accurately and reliably capture the level of SCI; it should be correlated with other measures of inflammation, without being overshadowed by any acute inflammation or other short-term influences. Second, it should be easily measured; stable over long time periods *in vitro* and *in vivo* (i.e., high test-retest reliability and temporal stability); and independent of smaller day-to-day variations, such as circadian/diurnal rhythm and fasting, while still being sensitive to significant contributions of psychosocial, environmental, and lifestyle factors as well as onset—or resolution—of chronic pathological processes.

### suPAR as a Biomarker for Systemic Chronic Inflammation

We propose that the blood levels of the protein *soluble urokinase plasminogen activator receptor* (suPAR) is a robust biomarker of SCI, with potential to be the new gold standard for measuring SCI. suPAR has been found to be a broad prognostic biomarker associated with incident disease and adverse clinical outcomes across both general and patient populations. suPAR has been reviewed as a marker of kidney disease, sepsis, cardiovascular disease, and inflammatory disorders (10–13)—but given its nonspecific associations with immune activity and prognosis in very diverse diseases and conditions, it is not a disease-specific diagnostic marker. We think it represents a common underlying disease-process shared by many diseases; that is, SCI (14).

We bring forward 10 properties and characteristics of suPAR supporting the argument that suPAR is a biomarker of SCI: (i) Expression and release of suPAR is upregulated by immune activation; (ii) urokinase plasminogen activator receptor (uPAR) and suPAR exert pro-inflammatory functions; (iii) suPAR is associated with the amount of circulating immune cells; (iv) Blood suPAR levels correlate with the levels of established inflammatory biomarkers; (v) suPAR is minimally affected by acute changes and short-term influences; (vi) Like SCI, suPAR is non-specifically associated with multiple diseases; (vii) suPAR and SCI both predict morbidity and mortality; (viii) suPAR and SCI share the same risk factors; (ix) suPAR is associated with risk factors and outcomes of inflammation above and beyond other inflammatory biomarkers; (x) The suPAR level can be reduced by anti-inflammatory interventions and treatment of disease.

Since no clear definition or criterion has previously been established for the state of SCI, we review the supporting literature, integrating evidence from many different sources and studies (including experimental, population-based, and clinical research), and propose a research agenda that can help test the hypothesis that suPAR indexes SCI.

### What Is suPAR?

**Figure 1:**
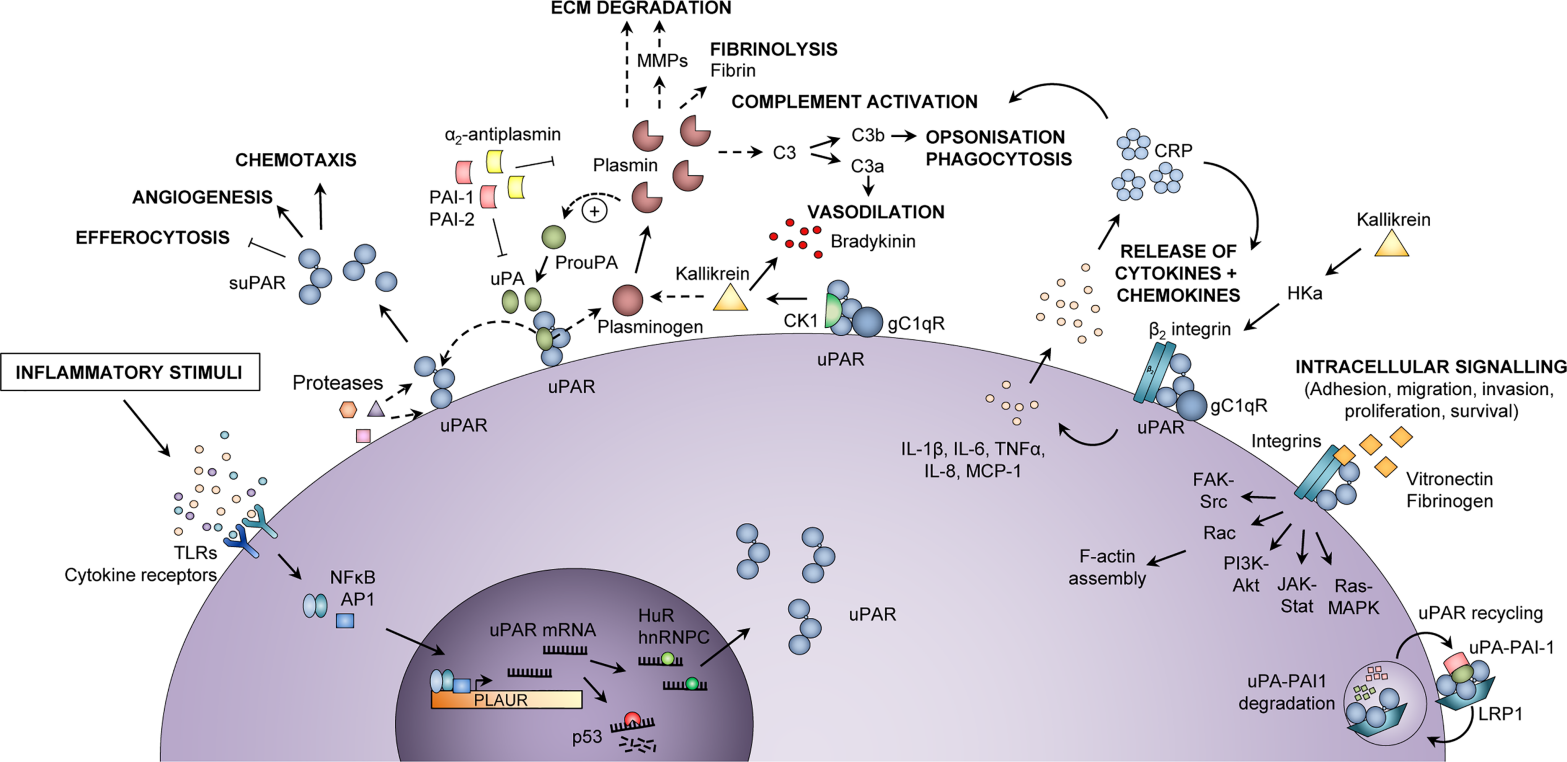
Inflammatory functions of uPAR and suPAR. Upon an inflammatory stimulus, e.g., stimulation of toll-like receptors (TLRs) or cytokine receptors, the expression of urokinase plasminogen activator receptor (uPAR) in immunologically active cells is increased *via* activation of transcription factors, such as nuclear factor kappa-light-chain-enhancer of activated B cells (NF-κB) and activator protein 1 (AP1), which bind to the promoter region of the *PLAUR* gene. The uPAR mRNA is either degraded (by p53) or stabilized for translation (by HuR or hnRNPC), after which uPAR is expressed at the cell surface, bound to the membrane *via* a glycosyl phosphatidylinositol (GPI) anchor. At the cell surface, uPAR can become cleaved by various proteases or its own ligand urokinase plasminogen activator (uPA), thus generating suPAR, which plays a role in inflammation by impairing neutrophil efferocytosis and stimulating angiogenesis and chemotaxis. Active uPA cleaves plasminogen to plasmin, which in turn cleaves and activates uPA. Plasmin activates matrix metalloproteases (MMPs), cleaves extracellular matrix (ECM) components, degrades fibrin, and activates the classical complement pathway, thereby promoting migration and invasion of cells, fibrinolysis, vasodilation, opsonization, and phagocytosis of foreign pathogens. Co-localization of uPAR with the proteins cytokeratin-1 (CK1) and globular C1q receptor (gC1qR) on the surface of endothelial cells also promotes vasodilation through release of bradykinin *via* activation of kallikrein. In a complex with β_2_ integrin and gC1qR, uPAR also induces release of cytokines (IL-1β, IL-6, TNFα) and chemokines (IL-8, MCP-1), upon stimulation by cleaved high molecular weight kininogen (HKa). Cytokines stimulate the production of C-reactive protein (CRP) from the liver, and CRP itself functions as an opsonin and also activates the classical complement pathway. Furthermore, uPAR interacts with vitronectin, fibrinogen, and integrins, mainly α_M_β_2_ integrin (Mac-1) but also β_1_ and β_3_ integrin complexes, activating intracellular signaling pathways that facilitate cell adhesion, migration, invasion, proliferation, and survival by affecting F-actin assembly and gene transcription. The activity of uPA and plasmin is inhibited by plasminogen activator inhibitor (PAI)-1, PAI-2, and α_2_-antiplasmin. Binding of PAI-1 and low-density lipoprotein receptor-related protein 1 (LRP1) mediates endocytosis of uPAR-uPA-PAI-1 complexes, followed by lysosomal degradation of uPA and PAI-1 and recycling of uPAR back to the membrane. In endothelial cells, co-localization of uPAR with CK1 and gC1qR activates kallikrein and promotes the release of the vasodilator bradykinin. hnRNPC, heterogeneous nuclear ribonucleoprotein C; HuR, Hu antigen R; IL, interleukin; MCP-1, monocyte chemoattractant protein-1; TNFα, tumor necrosis factor α. Adapted from Rasmussen, LJH (2018) (19) with permission.

The protein suPAR is found in blood [plasma, serum (15, 16)] and other body fluids [cerebrospinal fluid (17), saliva (18), urine (15)] and is the soluble form of the membrane-bound receptor uPAR. When expressed on the cell surface membrane, uPAR is a central mediator of plasminogen activation and fibrinolysis, and is involved in several critical cellular processes by regulating extracellular matrix degradation (**Figure 1**). As such, uPAR is involved in proliferation, migration, adhesion, angiogenesis, and in the inflammatory response (20). Proteolytic cleavage of uPAR releases the soluble form, suPAR, to the bloodstream (21).

Release of suPAR from immune cells is increased upon an inflammatory stimulus (**Figure 1**); thus, the blood suPAR level is thought to reflect an individual’s level of inflammation and immune activation (13). suPAR is indeed positively correlated with several inflammatory biomarkers, including CRP, IL-6, and TNFα (22–24), see **Appendix I**. While the suPAR level is elevated by a variety of pro-inflammatory conditions, it is generally low—although still detectable—in healthy individuals. In blood donors, the median suPAR level is around 2 ng/mL (25), and women generally have slightly higher suPAR than men (25–27). However, suPAR seems to increase more with age in men compared to women, and suPAR is similar among men and women in older adults (≥74 years of age) (28). suPAR concentrations are higher in serum than in plasma within individuals (27, 29).

A person’s suPAR level is determined by various factors, including genetics, lifestyle, and chronic- and acute disease. How much each of these factors contributes to suPAR has not been fully elucidated, but suPAR seems to capture the overall impact of these. In addition to adaptable contributions by lifestyle, underlying chronic disease, and acute conditions, genetic predispositions also affect an individual’s tissue uPAR expression and blood suPAR level. In a recent genome-wide association study, we found that blood suPAR levels were under substantial genetic influence (30), with a heritability estimate of 60% and 13 independently genome-wide significant sequence variants associated with suPAR across 11 distinct loci. Associated variants were found in and around *PLAUR* as well as the gene encoding the uPAR ligand urokinase plasminogen activator (uPA, or urokinase) *PLAU*, the kidney-disease-associated gene *PLA2R1*, and genes with relations to glycosylation, glycoprotein biosynthesis, and the immune response (30). This indicates that a combination of polymorphisms in different genes may affect the immune system and cause a higher basal level of suPAR. In studies of genetic polymorphisms in *PLAUR*, the polymorphisms rs344781 and rs4251923 have been associated with various clinical conditions (31–34), but none of these studies examined the corresponding suPAR levels and do therefore not offer a genetic explanation for increased suPAR levels in disease in general.

suPAR is removed from the circulation *via* renal excretion and cardiac clearance (27). Elevated suPAR levels are strongly associated with decline in kidney function, and as a result of poor filtration patients on dialysis have consistently been shown to have very high suPAR levels (35, 36). However, suPAR retains its prognostic value even at low glomerular filtration rates (37), indicating that it is not just a marker of kidney function.

#### Structure of suPAR

**Figure 2:**
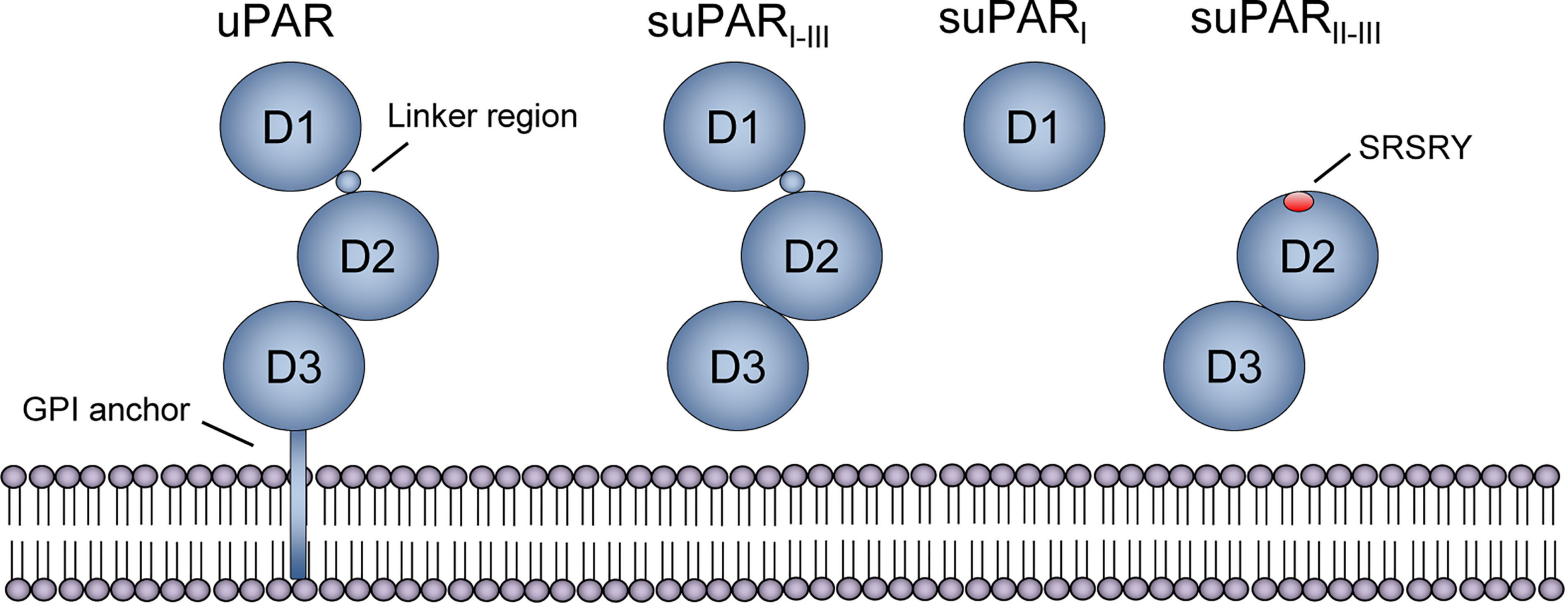
Structure of uPAR and suPAR isoforms. Soluble urokinase plasminogen activator receptor (suPAR) is the soluble form of the membrane-bound receptor uPAR, which is tethered to the membrane by a glycosyl phosphatidylinositol (GPI) anchor. The protein consists of three domains, D1-D3, that are connected with a linker region between D1 and D2D3. Several cleavage sites exist, both in the linker region and the GPI anchor, and proteolytic cleavage generates three suPAR isoforms: full-length suPAR_I-III_, suPAR_I_, and suPAR_II-III_. Cleavage of uPAR/suPAR in the linker region exposes an SRSRY sequence, which is involved in chemotaxis. Reprinted from Rasmussen, LJH (2018) (19) with permission.

uPAR and suPAR share the same overall structure, aside from the glycosyl phosphatidylinositol (GPI) anchor that tethers uPAR to the cell membrane. Both have three homologous domains, D1-D3, connected by a linker region between D1 and D2 (**Figure 2**). uPAR has cleavage sites for several proteases in the linker region (chymotrypsin, elastase, matrix metalloproteases, cathepsin G, plasmin, uPA) and the GPI anchor (phospholipase C and D, cathepsin G, plasmin), which upon cleavage can result in three suPAR isoforms (suPAR_I-III_ [full-length isoform], suPAR_I_, suPAR_II-III_) (21), **Figure 2**. The molecular weight of suPAR varies between 24–66 kDa due to variations in posttranslational glycosylation (21, 27). Additional isoforms generated by alternative splicing have been described on the RNA level, but whether these are transcribed and their possible roles remain unclear (38).

## 10 Properties and Characteristics Indicating That suPAR Is a Marker of SCI

### 1. Expression and Release of suPAR Is Upregulated by Increased Immune Activation

uPAR is mainly expressed on the cell membrane of immune cells, such as monocytes, macrophages, neutrophils, and activated T-lymphocytes, but also on endothelial cells, fibroblasts, vascular smooth muscle cells, megakaryocytes, keratinocytes, and some cancer cells (21). Expression of uPAR is normally low, but expression is increased during activation and differentiation of leukocytes, extracellular matrix remodeling, wound healing, stress, injury, inflammation, and in tumor cells and tumor-associated stromal cells of many cancers (20). Specifically, stimulation of innate immune receptors, e.g., toll-like receptors (TLRs) or cytokine receptors, on immunologically active cells induces expression of the gene encoding uPAR, *PLAUR*, *via* activation of transcription factors, including *nuclear factor kappa-light-chain-enhancer of activated B cells* (NF-κB) and activator protein 1 (AP1) (21), **Figure 1**. The uPAR mRNA is either degraded or stabilized for translation. After translation, uPAR localizes to the cell surface where it is bound to the cell membrane by the GPI anchor. At the cell surface, uPAR can be cleaved by its ligand uPA or other proteases, as mentioned above, thereby releasing suPAR to the bloodstream or other body fluids.

The specific inflammatory mediators shown to increase the expression of uPAR along with release of suPAR *in vitro* and *in vivo* include lipopolysaccharide (LPS), which increases the mRNA expression of uPAR *in vitro* (39, 40) and stimulates the release of suPAR (41). Injection of LPS in healthy human subjects has also been shown to increase the expression of uPAR on circulating monocytes and increase the blood levels of suPAR (42, 43). IL-8, TNFα, granulocyte-colony stimulating factor (G-CSF), and *N*-formyl-met-leu-phe (fMLP) have been shown to stimulate human peripheral blood mononuclear cells and neutrophils to rapidly increase surface expression of uPAR and induce release of suPAR (44, 45). Similarly, suPAR release from endothelial cells and vascular smooth muscle cells increases in response to IL-1β *in vitro* (46). In contrast, co-administration of IL-6 abolishes LPS-induced suPAR release (43).

In summary, increased immune activation and stimulation by inflammatory mediators induce the gene expression of uPAR and release of suPAR, *via* major inflammatory transcriptional pathways regulated by NF-κB and AP1, and increase the blood concentration of suPAR. This suggests that during states of inflammation, the expression of uPAR and suPAR will be increased.

### 2. uPAR and suPAR Exert Pro-Inflammatory Functions

Cell migration is important for inflammation and immune activation, and uPAR facilitates migration of immune cells through tissues, **Figure 1**. uPAR localizes its ligand uPA to the cell surface of immune cells, where active uPA cleaves plasminogen to generate the active protease plasmin (20). Plasmin, in turn, activates matrix metalloproteases (47) and cleaves a range of extracellular matrix components, degrades fibrin, and activates the classical complement pathway (generating the anaphylatoxins C3a, C4a, and C5a, and the opsonin C3b) (48). This promotes extracellular matrix degradation, activation of sequestered growth factors (20, 49, 50), cell migration and invasion, fibrinolysis, vasodilation, increased vascular permeability, opsonization, and phagocytosis of foreign pathogens.

Moreover, binding of uPA to uPAR facilitates non-proteolytic functions involved in cell migration. The interaction promotes clustering of uPAR in plasma membrane lipid rafts in the leading edge of migrating cells. It also increases binding of uPAR to the extracellular matrix protein vitronectin and to integrins (α_3_β_1_, α_5_β_1_, α_M_β_2_, α_v_β_3_) (20) and their extracellular matrix ligands (e.g., laminins, fibronectin, collagens, vitronectin). This interaction activates intracellular signaling pathways that promote cell adhesion, migration, invasion, proliferation, survival, and immune activity (20), **Figure 1**. These functions are in play during recruitment of monocytes to inflamed tissue, where complexes of uPAR and α_M_β_2_ integrin/Mac-1 expressed in leukocytes interact with intracellular Src kinases upon binding to vitronectin or fibrinogen, thereby regulating adhesion and cell migration of mononuclear cells (51). Thus, uPAR, uPA, and β_2_ integrin provide the adhesion/degradation interactions between immune cells and endothelial cells or extracellular matrix, required for leukocytes to invade inflamed tissue in response to a chemotactic signal (48). Additional mechanisms by which uPAR regulates inflammatory processes have been suggested (**Figure 1**). These include co-localization of uPAR with cytokeratin-1 (CK1) and globular C1q receptor (gC1qR) on the surface of endothelial cells, which promotes release of the vasodilator bradykinin. Another mechanism is the simultaneous stimulation of uPAR, β_2_ integrin, and gC1qR by cleaved high molecular weight kininogen, which induces release of cytokines (IL-1β, IL-6, TNFα) and chemokines (IL-8, monocyte chemoattractant protein-1 [MCP-1]) from blood mononuclear cells (52). All these processes contribute to sustaining the inflammatory response and to the cardinal signs of inflammation: swelling (tumor), redness (rubor), heat (calor), pain (dolor), and loss of function.

**Figure 3:**
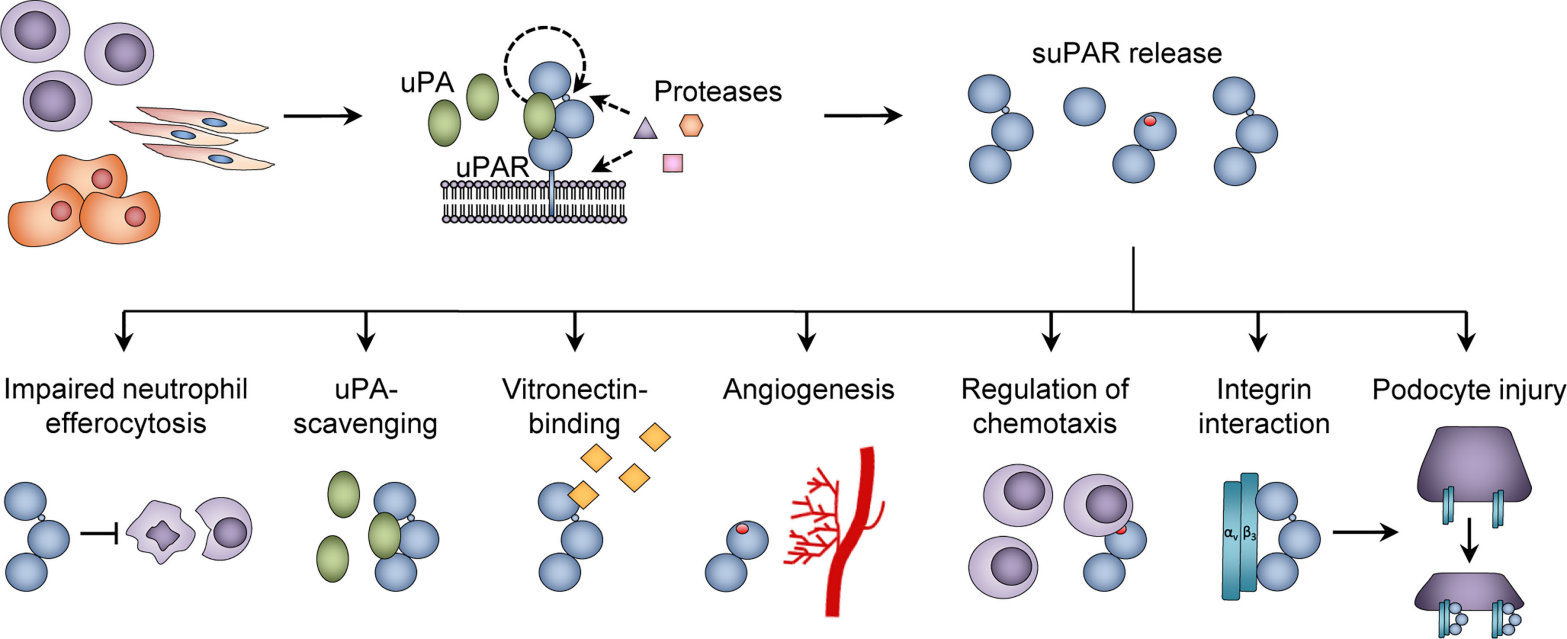
Functions of suPAR. The urokinase plasminogen activator receptor (uPAR) is expressed on the surface of immune cells, endothelial cells, and vascular smooth muscle cells, and proteolytic cleavage in the linker region or glycosyl phosphatidylinositol (GPI) anchor of uPAR generates soluble uPAR (suPAR). Various functions of suPAR have been proposed, including inhibition of neutrophil efferocytosis; binding of urokinase plasminogen activator (uPA) and vitronectin; stimulation of angiogenesis *via* endothelial sprouting and tube formation; promoting chemotaxis; and interactions with β_3_ integrin, which is suggested to cause podocyte injury in the glomeruli. Adapted from Rasmussen, LJH (2018) (19) with permission.

Although research into the active functions of suPAR has been limited, a number of immunological roles have been suggested. First, full-length suPAR_I-III_ is able to bind vitronectin (53) (**Figure 3**), to form a uPA-suPAR-vitronectin complex, which may allow vitronectin-directed activation of uPA at cellular surfaces or extracellular matrix sites (54). Second, suPAR_II-III_ may directly exert multiple pro-inflammatory functions by exposing an N-terminal SRSRY amino acid sequence (**Figure 2**, **Figure 3**). This SRSRY sequence acts as a chemotactic agent by interacting with the G protein-coupled receptor FPR-like receptor 1 (FPRL1) expressed on immune cells, including monocytes, lymphocytes, and neutrophils (44, 55, 56), and suPAR has also been shown to elicit cancer cell migration *via* this sequence *in vitro* (57). The SRSRY sequence is also involved in chemokine cross-regulation, preventing cell migration mediated by the chemokines MCP-1, CCL5, and fMLP (58). Additionally, the exposed SRSRY sequence stimulates angiogenesis with endothelial sprouting and tube formation, independent of uPA and vascular endothelial growth factor (59). Third, both suPAR and uPAR may impair phagocytic clearance of apoptotic neutrophils and other immune cells (**Figure 3**). This lack of neutrophil efferocytosis might contribute to sustaining the inflammatory response (60).

suPAR has also been ascribed some negative regulatory functions. The cleavage of uPAR into suPAR appears to abrogate uPA-mediated plasminogen activation, integrin-mediated intracellular signaling, and cellular migration (20). Full-length suPAR_I-III_ retains its ability to bind uPA through the D1 domain, and thereby acts as an uPA-scavenger (61), **Figure 3**. suPAR_II-III_ and suPAR_I_ are not able to bind and activate uPA or vitronectin, and cleavage of uPAR into these suPAR isoforms may comprise a form of negative regulation of plasminogen activator activity without affecting serum uPA levels (62).

Unlike stimulation with inflammatory cytokines, stimulation of whole blood with high concentrations of suPAR for up to 24 hours has minimal effect on the expression of inflammatory cytokines (IP-10, IL-6, IL-10, and TNFα) (63). This could explain why suPAR is allowed to circulate freely without immediately being cleared, in contrast to most inflammatory biomarkers that often exert strong local-acting effects on immune- and non-immune cells.

Finally, uPAR has been identified as a universal marker of senescent cells, and suPAR release from senescent cells is part of the senescence-associated secretory phenotype (SASP) (64, 65).

In summary, uPAR plays multiple important roles in the inflammatory response, including cell migration, invasion, proliferation, vasodilation, phagocytosis, as well as release of cytokines and chemokines. The full functions of suPAR remain unclear; there are indications that suPAR may mediate chemotaxis of immune cells, promote angiogenesis, and prevent neutrophil efferocytosis.

### 3. suPAR Is Associated With the Amount of Circulating Immune Cells

Many of the immune system’s functions are maintained by circulating immune cells. Inflammation is an essential mechanism of the innate immune system and part of the first line of defense against insults and infections.

suPAR is positively correlated with total white blood cell count (66, 67), and correlation analyses indicate that blood suPAR levels are associated with cells of both the innate and the adaptive immune response, **Appendix I**. Specifically, suPAR has been found to be correlated with innate immune cells including neutrophil count, monocyte count, and eosinophil count (66, 68, 69). For the adaptive immune system, suPAR has been correlated with lymphocyte count (69); however, other studies do not find a correlation between suPAR and lymphocyte count (66, 70, 71), which could suggest that suPAR is mainly associated with innate rather than adaptive immune cells. In line with this, uPAR expression is largely confined to pro-inflammatory monocyte subsets during the inflammatory response of acute liver failure (72) and to monocytes, neutrophils, and macrophages, but not lymphocytes, of patients with cirrhosis (41). LPS stimulation promoted the release of suPAR from monocytes, but not lymphocytes (42, 72).

In mice, suPAR has been found to originate from the expansion of uPAR-expressing bone marrow-derived immature myeloid cells (73). Myeloid expansion occurs under many clinical conditions, and, during inflammation, pro-inflammatory mediators—including cytokines (IL-1, TNFα, interferons [IFNs]), PAMPs, and DAMPs—regulate hematopoiesis and increase the myeloid output of bone marrow cells (74). A sustained overproduction of myeloid cells during SCI could result in increased suPAR levels in various conditions, e.g., chronic infections, autoimmune disease, and chronic inflammatory diseases, such as cardiovascular disease or type 2 diabetes.

In summary, suPAR is associated with the amount of circulating immune cells, mainly neutrophils and monocytes, and has been found to originate from expansion of myeloid lineage cells. This indicates that suPAR is associated with immune activity and could suggest that suPAR, like inflammation, is associated with innate rather than adaptive immune responses.

### 4. Blood suPAR Levels Correlate With the Levels of Established Inflammatory Biomarkers

The inflammatory response—acute as well as chronic—is mediated by numerous different cell types, inflammatory mediators (e.g., cytokines or chemokines), and their receptors.

Due to the lack of an operational definition of SCI, we are unable to assess suPAR’s internal consistency with other measures of SCI. However, suPAR is positively correlated with a multitude of biomarkers of inflammation. Plasma and serum suPAR levels have been found to be positively correlated with traditional markers of inflammation (see **Appendix I**), including CRP (23, 75–77), erythrocyte sedimentation rate (22, 78, 79), fibrinogen (80, 81), procalcitonin (23, 82), white blood cell count (68, 81), neutrophils (66, 68), monocytes (66, 69), and a number of cytokines and chemokines, e.g., IL-1β (22), IL-6 (83), IL-8 (CXCL8) (68), IL-10 (22, 68), IL-18 (84), MCP-1 (CCL2) (68), and TNFα (23, 24, 68).

But even though suPAR is positively correlated with established markers of inflammation, the correlations with many of these are weak (**Appendix I**). For example, reported correlations between suPAR and CRP range between 0.15–0.30 in population-based studies (69, 75, 76, 85), and between 0.15–0.53 (P<0.001) in clinical studies (71, 86). Similarly, the correlations with IL-1β (22), IL-10 (22, 68), erythrocyte sedimentation rate (22, 78), and white blood cell count (68, 81) were weak. The correlation of suPAR with other inflammatory markers generally appears to be stronger in patients with severe or exacerbated disease (68, 87, 88), maybe driven by a larger increase in suPAR levels related to presence of organ damage or dysfunction. When comparing suPAR to CRP, CRP is closer correlated with many of these inflammation markers, including erythrocyte sedimentation rate (89), fibrinogen, and IL−6 (69, 83). This difference suggests that CRP and suPAR reflect different aspects of inflammation, as previously described (90), and are not two measures of the same thing.

In summary, suPAR being positively correlated with established markers of (acute) inflammation, supports the role of suPAR as a marker of inflammation itself, although suPAR’s weaker correlation to acute phase proteins and cytokines compared to CRP suggests that suPAR may describe another type of inflammation.

### 5. suPAR Is Minimally Affected by Acute Changes and Short-Term Influences

Some of the most important pro-inflammatory cytokines have limited value as clinical biomarkers of SCI due to their short half-life, circadian fluctuations (IL-1, IL-12, TNFα, IFNγ), and susceptibility to variations in dietary intake (IL-6, TNFα), physical activity (IL-1 receptor antagonist [IL-1ra], IL-6, IL-10), and sample handling (91, 92). Furthermore, some cytokines, like IL-1β and TNFα, are even undetectable in healthy individuals with current commercially available assays (93, 94), or otherwise at or near the limits of accurate detection range, creating substantial variability and uncertainty in measured concentrations (95). These factors may obscure the detection and interpretation of clinically relevant changes in the concentrations of these inflammatory markers.

In contrast, suPAR is a very stable protein, which is subject to minimal circadian fluctuation (24, 27, 96, 97), it is readily quantifiable both in healthy (25) and sick individuals (98), and it maintains a steady sample concentration after repeated freezing/thawing cycles (27, 99). Thus, there are no requirements for special collection procedures or need for fasting blood samples. It also has low within-person variability and is stable in individuals with only small changes over time; it had an excellent intraclass correlation coefficient (0.91, 95% CI 0.88-0.93) over 4 months in healthy individuals (100) as well as a high intra-individual correlation in samples taken 5-7 years apart in population-based studies, with *r*=0.55 for suPAR measured at baseline and 5 years later in the Danish Inter99 Study (101), and *r*=0.58 for suPAR measured at age 38 and age 45 in the New Zealand Dunedin Study (102), significantly higher than log-transformed CRP levels (*r*=0.48) and log-transformed IL-6 levels (*r*=0.45) in the same study (103) (untransformed CRP levels were correlated at *r*=0.26 and untransformed IL-6 levels at *r*=0.39, unpublished data).

suPAR and CRP have previously been suggested to reflect different aspects of inflammation, with CRP being a marker of acute infection and metabolic inflammation, and suPAR being a marker of cellular inflammation and subclinical organ damage (90). Corroborating this theory, suPAR has been found to be differently related to cardiometabolic risk factors, for example, it is only weakly correlated with body mass index (BMI) (26, 69, 104), while CRP and BMI were strongly correlated (69). Furthermore, unlike CRP, suPAR was not correlated with body temperature, week of menstrual cycle, and use of anti-inflammatory medication (69).

Several studies have shown slower, delayed suPAR level increases in response to acute inflammatory stimuli compared to traditional inflammation markers. Knee surgery induced a significant increase in IL-6 and IL-10 between baseline and 1 day after surgery, while suPAR was unchanged the first day after surgery but had increased significantly 4 weeks after surgery (63). Similarly, patients admitted for myocardial infarction had continued rising CRP levels throughout the first 24 hours, while suPAR levels remained stable and unaltered (96), suggesting that the contribution of an acute event to suPAR levels is minimal. Indeed, among acute trauma patients, suPAR measured shortly after trauma was not associated with the severity of the trauma, but was higher in those who later died compared with those who survived (105). Thus, an acute event might not immediately affect the suPAR level substantially, but the basal suPAR level at the time of the event reflects the level of SCI which is associated with the outcome (106). One can therefore speculate that patients with higher SCI—as indicated by elevated suPAR—at the time of traumatic injury or surgery have lower capacity to withstand the immunological challenges and complications caused by the trauma or surgical procedure. This might also be the case for many other conditions, that is, patients with a high basal level of SCI have impaired abilities to manage and tolerate disease.

In summary, the temporal and kinetic stability of suPAR, in addition to the correlations with many of the established inflammatory biomarkers (**Appendix I**), suggest that suPAR reflects a more chronic aspect of inflammation.

### 6. Like Systemic Chronic Inflammation, suPAR Is Non-Specifically Associated With Multiple Diseases

SCI is not a disease-specific process confined to one single line of pathology. Rather, SCI is associated with diverse diseases affecting different organ systems, such as metabolic syndrome, type 2 diabetes, liver disease, cardiovascular disease, cancers, depression, autoimmune diseases, neurodegenerative diseases, sarcopenia, osteoporosis, and immunosenescence (2).

**Figure 4:**
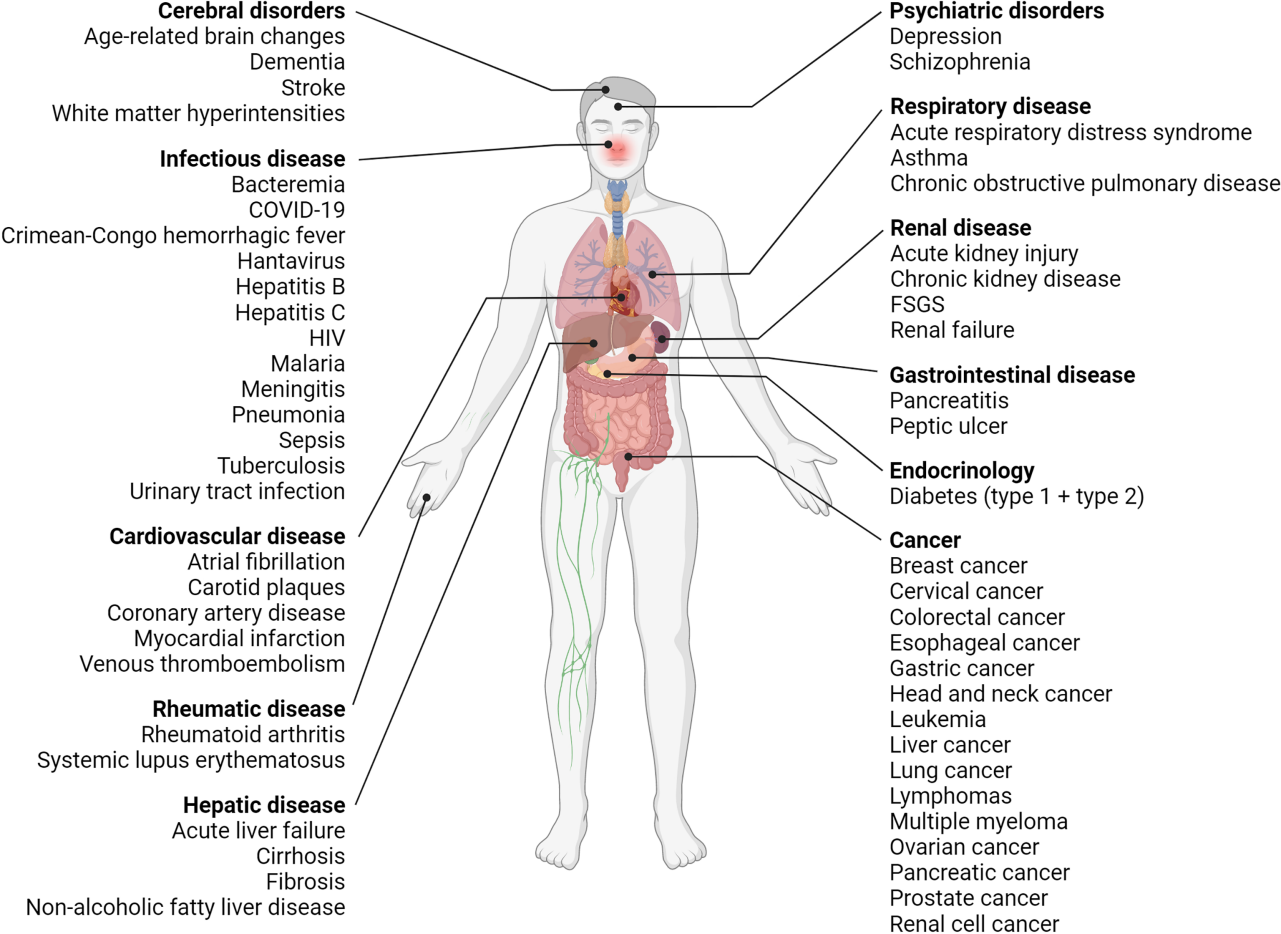
Overview of diseases with elevated suPAR levels. Clinical studies have shown that suPAR levels are elevated and associated with disease severity and prognosis in many diseases, including diseases of the brain, liver, kidneys, and respiratory system, cardiovascular disease, diabetes (type 1 and type 2), cancer as well as infectious, rheumatic, and psychiatric disorders. COVID-19, coronavirus disease 2019; FSGS, focal segmental glomerulosclerosis; HIV, human immunodeficiency virus; suPAR, soluble urokinase plasminogen activator receptor. Created with BioRender.com.

Likewise, increased suPAR levels are also associated with a wide range of diseases and disorders—non-communicable and infectious diseases alike, **Figure 4** and **Appendix II**. So far suPAR has been shown elevated in cardiovascular disease, including stroke, ischemic heart disease, venous thromboembolism, and incident atrial fibrillation (96, 107, 108); type 1 diabetes and diabetic complications (109, 110); incident and manifest type 2 diabetes (76, 111, 112); different types of cancer (see **Figure 4** and **Appendix II**) (15, 113–130); rheumatic disease, including rheumatoid arthritis (78, 79, 131) and systemic lupus erythematosus (22); asthma and chronic obstructive pulmonary disease (132); acute and chronic pancreatitis (115, 133); chronic liver disease, including non-alcoholic fatty liver disease and cirrhosis (68, 134, 135); incident acute kidney injury (136, 137) and chronic kidney disease (CKD) (138, 139); and dementia (77). As previously mentioned, suPAR is also elevated in infectious diseases caused by viruses, bacteria, and parasites, e.g., coronavirus disease 2019 (COVID-19) (37, 140), hepatitis B and C (135, 141), human immunodeficiency virus (HIV) (142), bacteremia (143, 144), meningitis (17, 145), urinary tract infection (88), pneumonia (71, 146), sepsis (23, 147), tuberculosis (148), malaria (149, 150), hantavirus (151), and Crimean-Congo hemorrhagic fever (87). suPAR is also elevated in pediatric disorders, including infections and CKD (71, 88, 139). Furthermore, suPAR may be associated with psychiatric disorders, including depression and schizophrenia (25, 152–154).

In summary, suPAR offers little diagnostic value, as it, like SCI, is elevated by many different diseases across multiple organ systems. It does however offer prognostic value.

### 7. suPAR and Systemic Chronic Inflammation Predict Morbidity and Mortality

SCI is predictive of disease development, progression, and mortality (2). Individual inflammation markers, composite scores including IL-6, CRP, TNFα, albumin, or neutrophil count, and high-dimensional inflammation measures have been found to be associated with morbidity and mortality (9, 155–157).

Elevated suPAR is associated with disease development, progression, severity, and risk of adverse outcomes. Thus, within and across various patient groups (**Figure 4**, **Appendix II**), high suPAR is associated with more advanced disease, exacerbations, and complications as well as presence of organ damage, comorbidities, and increased risk of adverse events and mortality (77, 158, 159). In critically ill patients, stably elevated or increasing suPAR levels were observed for non-survivors from the time of admission, while that of survivors remained stable or decreased until discharge (82, 146).

A suPAR value may therefore reflect the current health status of a patient, possibly by reporting the level of SCI and organ damage, and could contribute with valuable prognostic information in a clinical setting.

### 8. suPAR and Systemic Chronic Inflammation Share the Same Risk Factors

A number of risk factors are associated with increased levels of SCI, as recently reviewed by Furman et al. (2). These include higher age, chronic infections, tobacco smoking, physical inactivity, unhealthy diet, obesity, social isolation, psychological stress, and exposure to environmental or industrial toxicants. Moreover, early development and childhood circumstances have been shown to promote SCI in adulthood (2). The same risk factors have been shown to be associated with increased suPAR.

In the general population, suPAR increases with age (25, 76, 101, 102), not only with a person’s chronological age, but also with indicators of accelerated aging, such as faster rate of decline across multiple organ systems, older facial age, as well as physical and cognitive decline (102).

Chronic infections caused by viruses and bacteria are associated with elevated suPAR, which has been observed for hepatitis B and C (135, 141), HIV (142), and tuberculosis (148).

With regard to lifestyle, smoking is likely the most devastating cause of poor health, and the inhalation of smoke and toxicants are thought to activate the immune system through DAMPs. Studies in the general population have consistently shown that smokers have increased suPAR levels compared to non-smokers (26, 76, 102, 160), with smokers having around 1 ng/mL higher suPAR (101, 102). The effects of smoking on suPAR appear to be reversible to some degree such that ex-smokers have suPAR levels similar to that of never-smokers (101, 102). In a study of smoking cessation, daily smokers who were randomized to smoking cessation exhibited decreased suPAR levels that were no longer significantly different from that of never-smokers (66). In contrast, smokers and non-smokers did not differ in CRP levels, and smoking cessation had no effect on CRP levels (66).

Individuals with a sedentary lifestyle, unhealthy diet, or morbid obesity also have higher suPAR levels (26, 102, 161). The level of low-density lipoprotein (LDL) cholesterol (a major risk factor of cardiovascular disease due to buildups in the arteries) is positively correlated with suPAR, while high-density lipoprotein (HDL) cholesterol (which helps eliminate LDL cholesterol) is negatively correlated with suPAR (26, 76, 162). Exposure to toxicants like cadmium is also associated with increased suPAR (104).

Experiencing stressful life events—including relationship breakups, job loss, serious illnesses or accidents of self or close relatives, financial problems, being homeless or in jail, being physically or sexually assaulted, death of a friend or family member, and living through disasters—are associated with higher suPAR in midlife (103). In contrast, no associations were observed between stressful life events and CRP or IL-6.

Adult suPAR levels may have origins already in childhood. In two longitudinal birth cohort-studies, we showed that exposure to social and psychological risk factors during childhood—including adverse childhood experiences (such as abuse, neglect, and victimization), low childhood IQ, or poor childhood self-control—was associated with elevated suPAR levels later in life after controlling for adult BMI and smoking, but not with CRP or IL-6 (69, 163). In line with this, findings from previous research studying associations between childhood adversity and adult CRP, IL-6, and TNFα are inconsistent with several studies reporting non-significant associations (164).

In summary, suPAR is elevated in presence of well-established risk factors of SCI, including older chronological age, accelerated biological aging, chronic infections, smoking, physical inactivity, unhealthy diet, obesity, toxicants, and psychosocial stress-exposure during childhood and adulthood.

### 9. suPAR Is Associated With Risk Factors and Outcomes of Inflammation Above and Beyond Other Inflammatory Biomarkers

A new biomarker of SCI should be strongly and independently associated with outcomes of SCI above and beyond established inflammatory biomarkers and other widely available risk scoring systems.

Studies in various settings have shown that suPAR is indeed associated with risk factors as well as outcomes of SCI independently of common inflammation markers.

Elevated suPAR is associated with early-life risk factors and stressful experiences in childhood above and beyond CRP and IL-6 (69, 163). In our Environmental Risk (E-Risk) Study, we showed that children exposed to multiple forms of stress and violence during childhood and adolescence had elevated suPAR levels, but not CRP or IL-6, at age 18, even after adjustment for sex, BMI, and smoking (163). Moreover, participants exposed to cumulative adverse experiences across childhood and adolescence (domestic violence or multiple types of violence in childhood and adolescence) had elevated suPAR, but not CRP or IL-6. This underlines that measuring suPAR can be used to examine the health implications of stressful experiences in childhood beyond the established inflammation markers CRP and IL-6. We found that adverse experiences were prominent in the group of participants with low CRP and low IL-6 but high suPAR—a group of individuals who would have inadvertently been assigned to the low inflammation group if suPAR had not been assayed. Interestingly, we observed the strongest association between stress exposure and inflammation when combining the three biomarkers and thereby utilizing the potentially different inflammatory states they reflect.

The prognostic value of suPAR for various patient outcomes remains significant when controlling for CRP, including incident cancer, readmission, or mortality (77, 89). Of note, in patients with low CRP levels (<10 mg/L) suPAR still remained associated with mortality, further substantiating that suPAR does add prognostic value to CRP (77). In support of this, suPAR predicted all-cause- and cardiovascular disease mortality independent of CRP and IL-6 in a South African population (83).

**Figure 5:**
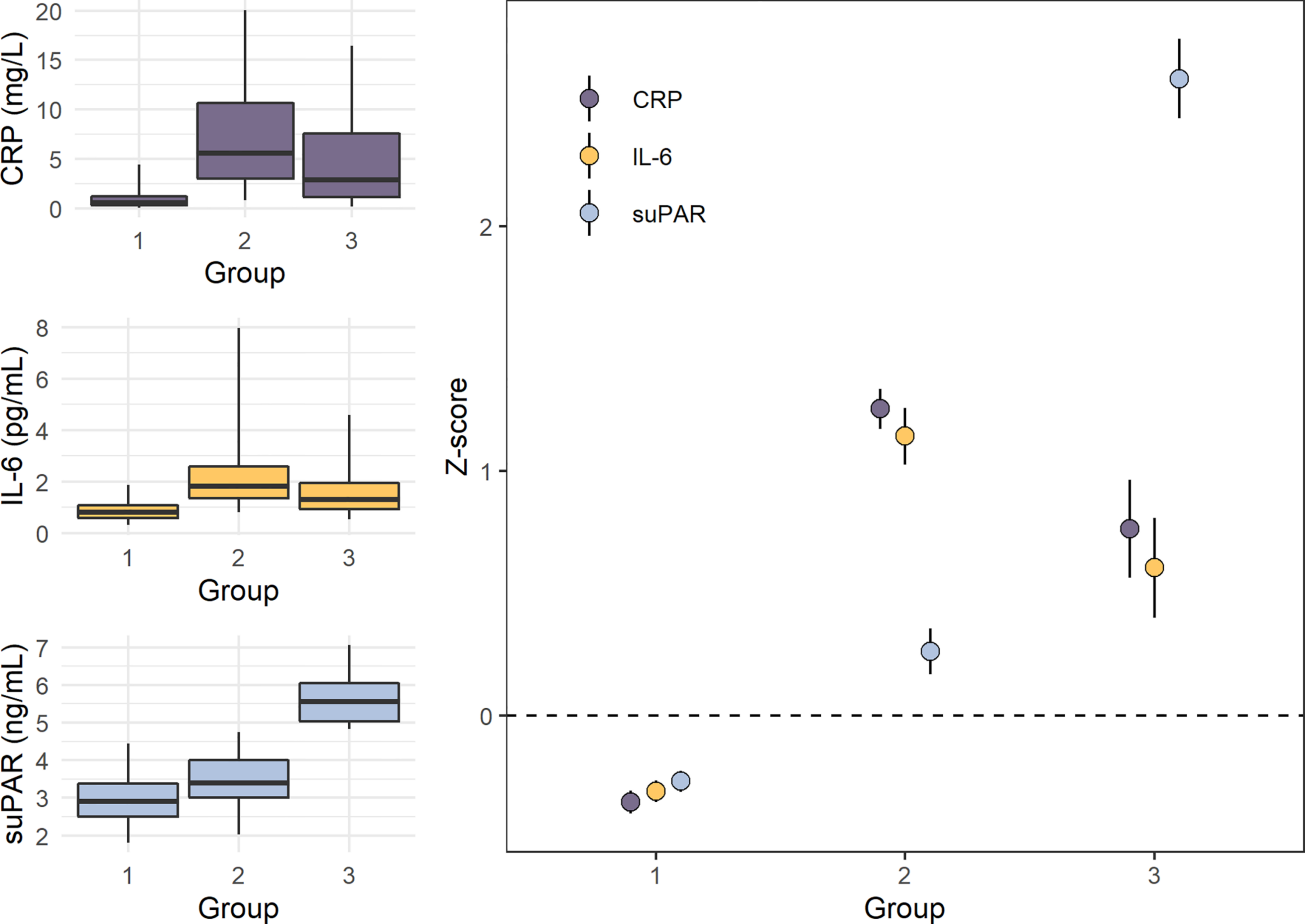
Levels of CRP, IL-6, and suPAR in the three inflammation groups identified by latent class analysis in the E-Risk study (n=1,390). Panels on the left show boxplots (box indicates median and interquartile range, and whiskers indicate 95% confidence interval) of untransformed C-reactive protein (CRP), interleukin-6 (IL-6), and soluble urokinase plasminogen activator receptor (suPAR) levels in the three groups, while the panel on the right shows mean Z-scores with standard deviations (M=0, SD=1). Group 1 (n=1,057) consisted of individuals with low CRP, IL-6, and suPAR. Group 2 (n=249) consisted of individuals with high CRP and IL-6 and moderately elevated suPAR. Group 3 (n=84) consisted of individuals with elevated CRP and IL-6 and high suPAR. Data from Rasmussen LJH, et al. (2020) (163).

Another remarkable finding from the E-Risk Study relates to the multidimensionality of inflammation and the utility of suPAR as a biomarker for indexing the chronic dimension of inflammation. We assessed the dimensionality of the three inflammatory biomarkers CRP, IL-6, and suPAR using latent class analysis in this cohort of healthy young adults, and we identified three latent groups of inflammation among participants in the cohort (**Figure 5**). Group 1 consisted of individuals with low CRP, low IL-6, and low suPAR. Group 2 consisted of individuals with high CRP, high IL-6, and moderately elevated suPAR. Lastly, Group 3 consisted of individuals with high suPAR, and moderately elevated CRP and IL-6 (163). We hypothesize that these groups represent three dimensions of systemic inflammation (low, acute, and chronic), identifying individuals with different types and levels of inflammation. Thus, Group 1 would represent low inflammation, Group 2 acute inflammation, and Group 3 SCI, with the key being high CRP/IL-6 as an indication of acute inflammation and high suPAR as an indication of SCI. For these 18-year old participants, members of Group 3 (high suPAR and moderately elevated CRP and IL-6) had been exposed to more adverse childhood experiences as well as victimization in childhood and adolescence, compared with those in Group 1 and Group 2 (163). These results need to be validated and tested in other and larger cohorts. Recently, a study of 574 adolescents reported similar findings, showing that persistent parent-child separation was more frequently observed among individuals with high suPAR and low CRP or with high suPAR and high CRP, compared to individuals with low suPAR and low or high CRP (165).

Adding suPAR level measurements to existing blood test panels would allow further stratification of inflammation type and level, and add valuable prognostic information above and beyond that of the current inflammatory biomarkers.

### 10. The suPAR Level Can be Reduced by Anti-Inflammatory Interventions and Treatment of Disease

Anti-inflammatory interventions to lower SCI can aim at targeting risk factors of inflammation, the inflammatory pathway, or diseases that are perpetuating chronic inflammation.

Lifestyle covers multiple risk factors for SCI, and altering lifestyle induces changes in suPAR levels. For example, change in diet or exercise has a positive impact by lowering the suPAR level (102, 166), and as mentioned above, smoking cessation results in a suPAR decrease (66), where former smokers can achieve comparable suPAR levels to that of non-smokers (26, 102). The resultant lowering of suPAR is also associated with lowering the risk of mortality (101), pointing to elevated suPAR as a modifiable risk factor.

Targeting the inflammatory pathway with anti-inflammatory medication has also been shown to lower suPAR levels; for example, suPAR levels were significantly lower after 14 days of corticosteroid treatment for acute exacerbation of COPD (80), after 2-4 weeks of glucocorticoid treatment in pediatric inflammatory bowel disease (167), and after 3-6 months of prednisolone treatment in HIV (168).

Other types of therapy, not aimed at the inflammatory response, have also been associated with reduced suPAR levels in various diseases. These include: long-term treatment with beta-blockers of patients with carotid stenosis (169); lipid-lowering treatment with statins of patients with aortic stenosis (170); surgical tumor resection (120, 123) or chemotherapy in treatment of cancer (15); treatment of acute exacerbation of COPD with bronchodilators, supplemental oxygen, or antibiotics (80, 81); treatment of community-acquired pneumonia with antibiotics (171); highly active antiretroviral therapy in HIV-1-infected patients (172); anti-malaria treatment in pediatric malaria (149); or even nutritional support during hospitalization with a high-protein diet for patients with systemic inflammatory response syndrome (173).

Thus, an increased suPAR level is modifiable and reversible, both by means of lifestyle changes or therapy targeting either disease or inflammatory state. Together, this makes suPAR valuable in assessing the effect of potential interventions aimed at reducing SCI as well as in measuring the risk imposed by an individual’s level of SCI.

## Discussion

In summary, this body of research provides strong support for the hypothesis that suPAR is a biomarker of SCI. suPAR is upregulated and released to the bloodstream from innate immune cells in response to increased immune activation. It serves inflammatory functions in itself and is positively, although often weakly, correlated with established inflammatory biomarkers, including CRP. suPAR has high stability as a biochemical analyte (temporal stability and low method-specific variance) and is minimally affected by short-term influences, circadian rhythm, and minor acute events. It shares risk factors and outcomes with SCI independently of other inflammatory biomarkers. Finally, suPAR possesses features that are important for a clinical prognostic biomarker (174): it reflects ongoing pathogenic processes with the ability to predict incident (76, 107, 158) or prevalent disease (77, 81, 116, 117, 121, 139), extent or severity of disease (68, 123, 125, 135), and risk of recurrence (96, 175) or fatality (76, 98, 147, 176); the blood concentration of suPAR is significantly altered in response to anti-inflammatory interventions, disease, or remission (80, 81, 149, 172); and it is readily quantifiable both in healthy (25) and sick individuals by means of safe and easy testing. Aside from a potential to index and assess a person’s level of SCI, suPAR can be a useful biomarker in the clinic. The stable kinetics of suPAR limits its value in monitoring immediate clinical responses to treatment of acute disease, but suPAR offers clinical value as a prognostic tool for clinical endpoints, due to its strong association with disease severity.

Together, this suggests that suPAR could be the best single marker of SCI, organ damage, and physiologic reserve, contributing added information about the systemic chronic inflammatory state to that of the commonly used (primarily acute) inflammatory markers. suPAR is not specific to any one disease in particular, but is elevated and has a strong prognostic value across many different disorders. As such, suPAR has limited value as a diagnostic tool. The broad associations across diseases suggest that blood suPAR levels reflect a shared feature of disease, which could very well be SCI. The recent studies that link elevated adult suPAR levels with psychosocial stressors in childhood and adolescence (69, 163) as well as physical and cognitive decline and accelerated biological aging (102) further contribute to our understanding of suPAR as a marker of chronic influences and mark the transition from viewing suPAR as a clinical biomarker associated with illness, to being a broader marker of underlying immune activity associated with early development, psychosocial stress, and accelerated aging, before the onset of disease.

Despite the consensus that SCI is health-damaging and constitutes a major risk factor for many diseases, the lack of good stable biomarkers reflecting SCI has so far left this an undiagnosable condition. This greatly limits not only research into chronic inflammation, but also poses a serious problem for treating patients, as emerging disease processes may go unnoticed, leading to development of manifest disease and detrimental health complications. Using suPAR as a measure of SCI has the potential of improving the estimation of a person’s underlying inflammatory burden and provide accurate assessments of interventions aimed at reducing inflammation, creating a valuable window of opportunity for treatment and prevention.

### A Marker of Diminished Immunological Capacity?

Based on the research and clinical findings on suPAR in disease reviewed here, the evidence points to suPAR as a quantitative indicator of a person’s level of SCI. In other words, the suPAR level mirrors the current level of underlying immune activity and the individual’s health state.

As described earlier, the suPAR level measured immediately after an acute trauma is not associated with the severity of trauma while still associated with survival during follow-up (105), suggesting that suPAR is not rapidly released as part of the acute response, which could, in turn, suggest that suPAR does not have an active, functional role in the immune response to acute events. Rather, suPAR may be an indicator of the immunological capacity of a person. That is, the level of SCI, as reflected by the suPAR level, indicates how well a person will tolerate and handle an immune challenge, with individuals with persistently elevated SCI having a lower capacity to withstand and manage injuries, trauma, or disease.

The strong prognostic value of suPAR in infectious diseases, with high suPAR being associated with a higher risk of adverse outcomes, could indicate that the higher the degree of SCI, the less efficient the immune system is at protecting the individual. In patients with COVID-19, an early elevation of suPAR is an indicator of poor prognosis, including increased risk of developing respiratory failure (140), acute kidney injury, and mortality (37). As a result, suPAR has been used to stratify COVID-19 patients in the Emergency Department, where patients with suPAR levels above 6 ng/mL were treated with the IL-1 receptor antagonist anakinra, which significantly reduced time to recovery and lowered mortality compared to the standard of care (177, 178). This raises the question of whether patients with elevated SCI (or suPAR) in general, and not only COVID-19 patients, will benefit from anti-inflammatory treatment through reduced morbidity and mortality.

Immunosenescence is a multifaceted decline in immune effectiveness, resulting in increased susceptibility to infections and age-related inflammatory diseases, diminished vaccine responses, and lower capacity to mediate anti-cancer responses and control tissue homeostasis and repair (179–182). Immunosenescence is characterized by age-related low-grade SCI (*inflammaging*), diminished response to new antigens, and the accumulation of memory T and B cells with a decrease in naïve cells (180). Given that high suPAR is associated with a lower effectiveness of the immune system, as illustrated by the elevated risk of disease progression and adverse events, as well as its associations with inflammation and multiple indicators of accelerated aging and functional decline, suPAR may be a useful biomarker of SCI that can be used to quantify the level of inflammaging and immunosenescence. In support of this, we recently reported elevated suPAR levels in a patient population characterized by accelerated aging and multiple signs of immunosenescence, including reduced capacity to respond to immune stimulation, defects in NF-κB signaling, and higher levels of inflammatory biomarkers (CRP, IL-6, IL-18, TNFα, growth differentiation factor 15 [GDF15]) and NLRP3 inflammasome expression compared to age-matched and young healthy controls (183).

### Difference From CRP

The most widely used biomarker of inflammation is the acute-phase reactant CRP. CRP and suPAR differ in their respective susceptibility to acute and chronic stressors, temporal specificity (timing of release) and response kinetics (speed, amplitude, and stability of release), and to the type of pathologies that they are most strongly related to.

CRP shares numerous of the 10 characteristics we propose for suPAR. Expression of CRP is induced by IL-6 and other cytokines (IL-1β, TNF) *via* NF-κB and other transcription factors as part of the acute-phase response or during inflammatory conditions and infections (184). CRP exerts a functional role in the inflammatory response, through activation of the classical complement pathway, induction of phagocytosis, apoptosis, and release of pro-inflammatory cytokines, as well as chemotaxis and recruitment of leukocytes to areas of inflammation (184, 185). CRP is primarily synthesized by hepatocytes, but it can also be produced by other cell types, including leukocytes, endothelial cells, smooth muscle cells, and adipocytes (185). The blood concentration of CRP correlates with the concentration of various cytokines, including IL-6 and TNFα. CRP is non-specifically associated with multiple diseases, and elevated CRP levels are associated with increased risk of incident disease and mortality (7). Many factors are associated with baseline CRP levels, including age, sex, lifestyle, blood pressure, and in particular, metabolic risk factors such as elevated blood lipids and obesity (185). Lifestyle interventions to reduce cardiovascular risk have been associated with lower CRP levels, and, in case of disease, treatment of the underlying pathology that is causing an acute-phase stimulus can reduce the CRP levels (7).

In contrast to IL-6, TNFα, and other cytokines (186), CRP is also not subject to diurnal variation (187) and does not respond to acute psychological stress challenges (188). CRP increases rapidly in response to acute stimuli, such as severe tissue damage, trauma, or acute infection. Some bacterial infections can dramatically increase CRP levels up to 1,000-fold in the span of 24–72 hours (189). When an inflammatory stimulus is terminated, the CRP level quickly decreases with a half-life of about 19 hours (189). As such, CRP is an excellent biomarker of bacterial infections and the acute inflammatory response. In contrast, the suPAR response to acute stimuli is much slower and the fold-change markedly smaller, e.g., around 1.3-fold increase in suPAR 7 days after diagnosis of ventilator-associated pneumonia and sepsis (146) or cardiac arrest (190). Compared to CRP, suPAR is an inferior diagnostic marker for discriminating between infections of bacterial vs. non-bacterial origins (191). Although suPAR has been found to be significantly elevated in critically ill patients, including patients with sepsis, compared to healthy controls, the ability of blood suPAR levels to discriminate sepsis from non-sepsis patients was poor compared to that of CRP (23).

Moreover, elevated CRP levels are associated with increased risk of incident disease, such as diabetes and cardiovascular disease, and mortality (192, 193), and CRP has been recommended as an adjunct screening tool for cardiovascular risk prediction in the general population (7). However, there is significant short-term within-person variability in CRP levels in the general population (194, 195), with approximately one-third of persons with elevated CRP levels (≥10 mg/L) being reclassified after repeated testing 2.5 weeks later (194). The variation was particularly high at higher CRP values—the cases in which clinicians are most likely to intervene. This variability in CRP means that using a single CRP measure to index SCI may lead to substantial misclassification (194). Moreover, a common approach in research studies that are investigating risk factors of SCI is to systematically remove all observations with CRP >10 mg/L to exclude participants with acute illness; however, this could introduce bias by also excluding individuals with actual SCI and high CRP (196).

For psychosocial stressors, several different types of adverse childhood experiences, including child maltreatment, bullying, and sexual abuse, have been associated with increased CRP levels. In addition, low socioeconomic status has been shown to be associated with higher CRP (197). However, findings are not consistent; several studies report non-significant associations, and in many cases, associations do not survive control for the confounding effects of BMI or smoking (164, 197). In a meta-analysis investigating associations between socioeconomic status and inflammation, only studies that did not control for BMI or smoking showed significant associations between CRP and socioeconomic status (197). Similarly, CRP and IL-6 did not show consistent associations with adult stressful life events, in contrast to suPAR (103). An explanation could be that these traditional biomarkers of inflammation may mix chronic and acute effects.

The variance in suPAR that can be ascribed to CRP is around 15–30% in general and healthy populations (**Appendix I**). As the two biomarkers appear to identify different classes of people at risk, using suPAR in combination with CRP can provide valuable information about an individual’s state of health.

### Passive Bystander or Active Disease Agent?

Whether suPAR plays an active role in disease development or is merely a passive bystander that reflects ongoing disease processes remains unresolved. A causal role of suPAR has been described in CKD, primarily in focal segmental glomerulosclerosis (FSGS).

Blood suPAR level is elevated in two thirds of patients with FSGS, and high blood suPAR concentrations induce renal injury in experimental models (175), and infusion of suPAR in uPAR-knockout mice induced proteinuria (175, 198). Moreover, the declining kidney function, which is associated with a high-risk genotype of the gene for apolipoprotein 1 (*APOL1*), is dependent on high plasma suPAR levels (199). This suPAR-induced renal injury is further dependent on suPAR’s interaction with β_3_ integrin (175), and the suggested pathological mechanism is a synergistic suPAR- and apolipoprotein 1-mediated activation of α_v_β_3_ integrin on the podocyte membrane (**Figure 3**), causing renal injury through podocyte foot process effacement, cell detachment, and disruption of the glomerular barrier with resultant proteinuria (199). The pathological suPAR production was caused by expansion of uPAR-expressing immature myeloid cells, which lead to increased suPAR levels and proteinuria in mice (73). Moreover, in uPAR knock-out mice, uPAR expression in transplanted hematopoietic cells was necessary for suPAR production and development of proteinuria (73).

As myeloid expansion occurs under many conditions without necessarily afflicting renal damage, it appears to be the combination of suPAR and high-risk variants of the *APOL1* gene that triggers CKD, and not just the presence of suPAR alone. During inflammation, pro-inflammatory mediators regulate hematopoiesis and increase the myeloid output of bone marrow cells (74). As suPAR is produced from myeloid cells, this chronic overproduction of myeloid cells could be a potential source of increased suPAR, not only in the pathogenesis of CKD, but also in conditions of SCI related to aging or disease, e.g., chronic infections, autoimmune disease, and chronic inflammatory diseases, such as CVD or type 2 diabetes.

If suPAR plays a causal role in CKD, and possibly in the pathogenesis of other diseases, inhibition or removal of suPAR could have a stabilizing effect on disease. Interestingly, renal disease was stabilized or even abrogated when lowering circulating suPAR levels, either through removal of suPAR with plasmapheresis, or by interfering with the suPAR-β_3_ integrin interaction using blocking antibodies or small molecule inhibitors (175). It would potentially have major clinical implications, if diseases with elevated suPAR could be treated *via* reduction of suPAR levels, and would suggest that suPAR was not just a passive by-product of uPAR signaling but could have an active role, at least in kidney disease. Experiments are ongoing to further document this causal role of suPAR, with some conflicting evidence (200). Whether increased suPAR levels in other diseases merely reflect the expression and activity of uPAR remain unknown.

As previously mentioned, suPAR is not dramatically upregulated in response to acute events and it is detectable in the blood even during states of normal homeostasis in healthy individuals, in contrast to most active inflammatory mediators. This could be speculated to indicate that suPAR might be less functionally active and therefore allowed to circulate freely without being rapidly cleared from the blood. However, a large buildup of suPAR in the blood over longer time could potentially create toxic concentrations that are inflicting the damage observed in kidney diseases like FSGS. As suPAR is removed from the blood by renal clearance, this mechanism could be particularly exacerbated in patients with poor kidney function.

### Research Agenda

While the existing evidence points to suPAR as a potential biomarker of SCI, there are still several questions to answer. In the following, we describe a research agenda with the purpose of improving the understanding of the link between suPAR and SCI as well as paving the way towards clinical implementation of suPAR.

First, studies should be designed to test the hypothesis that suPAR can actually be used to distinguish acute from chronic systemic inflammation. As previously described, we used latent class analysis in the E-Risk Study of CRP, IL-6, and suPAR, and identified three groups (**Figure 5**). Group 1 consisted of individuals with low CRP, low IL-6, and low suPAR. Group 2 consisted of individuals with high CRP, high IL-6, and moderately elevated suPAR. Group 3 consisted of individuals with high suPAR, and moderately elevated CRP and IL-6 (163). As mentioned, we think these results likely represent a method for determining a person’s level and type of inflammation, with Group 1 representing low inflammation, Group 2 acute inflammation, and Group 3 SCI, with suPAR being the indicator that differentiates acute inflammation from SCI. The observation that Group 3 had a higher proportion of traumatic childhood experiences indicates that suPAR levels reflect the long-term health effect of chronic stress that is not sufficiently identified by the established inflammation markers CRP and IL-6. These results need to be tested and validated in other and larger prospective cohort studies, using descriptive statistics to characterize any differences between the three groups and testing associations with both risk factors and long-term outcomes of SCI, such as chronic diseases and early mortality. If Group 3 is in fact characterized by SCI, we would expect to find stronger associations for this group with factors related to SCI. Furthermore, it should be tested if the prognostic information carried by using the composite measure with all three biomarkers can be reasonably approximated using only suPAR. We are currently planning international multi-cohort studies with longitudinal data to replicate and test this model.

Second, mechanistic studies are needed to map the molecular biology behind and the pathways leading to increased suPAR. As research indicates that suPAR is associated with innate immune cells, the association between suPAR and innate immune mechanisms should be further explored to elucidate whether suPAR shares pathways with other known drivers of chronic inflammation, e.g., the inflammasome (201). Testing the hypothesis that high suPAR represents a measure of lower immunological capacity or immunosenescence could be done by comparing individuals with high vs low suPAR in regard to their baseline levels of immune cell subset composition (e.g., ratios of memory:naïve and CD8:CD4 T cells), antibody levels, chronic infection status, as well as ability to elicit an immune response upon stimulation of isolated immune cells as measured by fold-change in cytokine production or immune cell signaling (e.g., STAT and NF-κB pathways). In addition, uPAR is induced during cellular senescence and released as suPAR as part of the SASP; the role of uPAR and suPAR in senescence of immune cells and the link to immunosenescence should be further explored.

Third, intervention studies aimed at lowering SCI or at preventing outcomes of SCI should use suPAR either as an effect measure, or to identify the target group for the intervention. Therefore, studies aimed at lowering SCI could test interventions that target risk factors of systemic inflammation and use suPAR as an effect measure to assess whether various lifestyle (e.g., smoking cessation, caloric restriction, physical activity), psychosocial, or clinical interventions have a positive effect on health by lowering the suPAR level. For example, randomized studies of social interventions in high-risk individuals could use suPAR to inform on the effect on health risk. Studies aimed at preventing outcomes of SCI in general populations could use suPAR levels to identify individuals with elevated SCI, either by using suPAR alone (e.g., suPAR >4 ng/mL in general populations), or by using suPAR along with CRP and IL-6 to identify people with inflammation levels consistent with the SCI group identified with latent class analysis (163). Individuals with high suPAR could then be randomized to an intervention or control group, to test whether individuals in the intervention group experienced a positive effect of the intervention on other health-related outcomes. For example, psychosocial interventions that reduce people’s psychological distress following trauma or other stressors might be able to reduce inflammation and improve people’s health as a result (202), and it should be tested if such interventions can also reduce suPAR levels. Studies aimed at preventing outcomes of SCI in patient populations could randomize patients with high suPAR to interventions that accelerate the diagnostic or treatment procedures, or to novel therapies. Possible interventions for patients with unexpected high suPAR could be referral to a fast-track cancer diagnostics program like the Diagnostic Outpatient Clinics (89), treatment with anti-inflammatory medications like the IL-1 receptor antagonist anakinra (178), or the use of screening with a multiple rule-out CT scanning. This approach was recently shown to be feasible in Emergency Department patients selected based on their prognosis (moderate-to-high risk patients based on the vital sign-based National Early Warning Score) rather than their specific symptoms (203). In that study of 100 patients from the Emergency Department, scanning patients with moderate-to-high risk according to their vital signs led to change in treatment or additional examinations in 37 patients, of which 24 were diagnostically significant, including change in acute treatment in 11 patients and identification of previously unrecognized malignant tumors in 10 patients (203). This intervention might similarly be feasible to test in patients in the Emergency Department presenting without specific symptoms and with high suPAR (>6 ng/mL) for whom the risk of severe disease and mortality is high (77) and the concern regarding radiation exposure is outweighed by the potential benefits of diagnosing a serious illness.

Fourth, establishment of cut-offs and clinical guidelines remains an important task for the successful implementation of suPAR analysis in healthcare settings. These will depend on the context, with certain cut-offs indicating SCI along with risk of incident disease in healthy or general populations, while higher cut-offs could be used to indicate risk of different adverse outcomes for specific clinical populations. For example, it has been suggested for patients in the Emergency Department that suPAR levels <4 ng/mL indicate that it is safe to discharge the patient (given that the patient does not have other acute indications), whereas levels >6 ng/mL should be considered as an alarming sign of risk for unfavorable outcomes, and levels >12 ng/mL are associated with a high risk of 28-day mortality (204). Direct testing of the benefits of using suPAR above 6 ng/mL for risk stratification is needed. In general populations, broader anti-inflammatory interventions targeting lifestyle behaviors or social risk factors—or even use of mild anti-inflammatory drugs—could be employed for people with elevated suPAR. In patient populations, the nature of an intervention would depend on the underlying diagnosis, made based on other clinical, biochemical, and physiological parameters. Given suPAR’s lack of disease specificity, it is not possible to establish one single clinical intervention for patients with high suPAR. A high suPAR level can direct attention to the patient and provide additional characteristics of the underlying health state as well as the extent and severity of disease. Similar recommendations could be made for patients with high suPAR as those made by the Sepsis-3 task force for Intensive Care Unit (ICU) patients with positive Quick Sequential [Sepsis-related] Organ Failure Assessment (SOFA) score, prompting further investigation for organ dysfunction, initiation or escalation of therapy as appropriate, and assessment of need for critical care or increased frequency of monitoring (205).

Fifth, large-scale omics-based approaches could provide further information on suPAR’s role in health and disease (206). Thus, integrating suPAR along with proteomic data on inflammatory biomarkers in a systems biology approach to explain SCI and related disease outcomes could identify novel direct and indirect interactions of suPAR with other inflammatory components. With this approach, suPAR emerged as one of the most important markers among 50 plasma proteins in a proteomic panel predicting acute myocardial infarction (207). However, other proteomic-based approaches have had limited ability to correlate suPAR levels and clinical outcomes in contrast to traditional ELISA-based detection methods (208, 209); even for ELISA methods, marked variation has been reported between assays (208, 210). The discrepancy between assay types could relate to different detections of suPAR isoforms, complexes of suPAR with its ligands (e.g., uPA, vitronectin) (210), or general proteomics-related challenges, such as cross-reactivity and non-specific interactions (211). This highlights the importance of understanding assay-related differences for suPAR to ensure robust prognostic capability, as the selection of assay for suPAR measurement could have direct impact on the clinical results obtained.

### Implications

Identifying suPAR as a new biomarker of SCI has implications for theory, for methods, for research, and for prevention.

For theory, this hypothesis offers a new way to characterize and define the state of SCI, which has long been acknowledged to be poorly understood (2, 5). Moreover, it contributes with a new understanding of suPAR as a biomarker, with high suPAR levels indexing presence of SCI along with increased risk of SCI-related outcomes and lower immunological capacity, i.e., the ability to tolerate and deal with incoming challenges to the immune system, such as physiological stress and disease. Additionally, the findings on early life risk factors and elevated adult suPAR provide support for the existing theory that the foundation for SCI in adulthood is laid already in childhood, with a wide variety of early-life risk factors having detrimental effects on life-long health through increased inflammation. Finally, the findings of suPAR being associated with inflammation as well as multiple indicators of accelerated biological aging and functional decline support the existing theories of immunosenescence and inflammation in aging, while also providing a new theory of suPAR being a clinically useful marker of SCI that can be used to quantify the level of inflammaging, and maybe even immunosenescence.

For methods, the measurement of blood suPAR levels comprises a new method to indicate the presence and to quantify and monitor the level of SCI and, with this, to detect and quantify the impact of various risk factors including social and psychological factors on health. Similarly, differentiating individuals in the three inflammation groups described by the E-Risk latent class analysis provides a new way to measure a person’s level and type of inflammation (163). Detecting and quantifying SCI further allows for the development of a clinical diagnosis of SCI using elevated suPAR as the main indicator. Being able to diagnose an individual with SCI will enable the identification of individuals at highest risk of developing manifest disease among previously undiagnosed individuals, and provide a more exact assessment of the level of the inflammatory burden of an individual along with any subclinical organ damage and immunological capacity (or lack thereof). This essential information will be missed by relying on markers of acute inflammation. Using suPAR as an adjunct to traditional inflammatory biomarkers provides the most information and allows for the distinction between types of inflammation, such as acute vs. SCI.

For research, suPAR can be used as a quantifiable intermediate outcome between early risk factors and more distal outcomes such as disease development or mortality, as suPAR is associated with early life, social, behavioral, environmental, and pathological risk factors as well as with poor health, accelerated aging, incident and prevalent disease, and mortality. Thus, future intervention studies can use suPAR as an effect measure to assess the effect of social, behavioral, or clinical interventions on health, without having to wait for many years for traditional health outcomes (e.g., disease) to develop, for the impact of the intervention to show. For example, randomized clinical trials of anti-inflammatory or anti-aging interventions intended to slow the course of aging could include suPAR as an outcome measure of SCI. In addition, suPAR could be used to study how stressful experiences become biologically embedded to affect a person’s downstream health, or it could be added as a new measure of long-term inflammatory processes to studies that investigate the causes of aging-related illness and the opportunities to improve health throughout the lifespan.

For prevention, since suPAR predicts future disease development, measuring suPAR creates an opportunity for prevention by targeted interventions among people with the highest risk of adverse health outcomes, as exemplified with the suPAR-guided anakinra treatment in COVID-19 (177, 178). Thus, suPAR analysis in various clinical settings, including assessment of socially vulnerable individuals, could inform imminent serious health risks.

In conclusion, there is cumulating evidence that blood suPAR levels represent a common underlying disease-process shared by many diseases; that is, SCI. We propose that suPAR is a robust measure of SCI with the potential of becoming a gold standard for assessing SCI in research and clinical settings.

## Author Contributions

LR has written and conceptualized the article. JP and JE-O have contributed to the writing and critically revised it. All authors contributed to the article and approved the submitted version.

## Funding

LR is supported by an international postdoc fellowship from the Lundbeck Foundation (grant no. R288-2018-380). JP is supported by an international postdoc fellowship from the Alfred Benzon Foundation (grant no. ABF-2018-91). Preparation of this manuscript was supported, in part, by research grants from the National Institute on Aging (AG032282 and AG049789), National Institute of Child Health and Human Development (HD077482), and Medical Research Council (MR/P005918/1, G1002190). The funders had no role in the conception, writing, or preparation of this manuscript, nor in the decision to publish.

## Conflict of Interest

JE-O is a named inventor on patents on suPAR as a prognostic biomarker. The patents are owned by Copenhagen University Hospital Amager and Hvidovre, Denmark, and is licensed to ViroGates A/S. JE-O is a co-founder, shareholder, and CSO of ViroGates A/S.

The remaining authors declare that the research was conducted in the absence of any commercial or financial relationships that could be construed as a potential conflict of interest.

## Publisher’s Note

All claims expressed in this article are solely those of the authors and do not necessarily represent those of their affiliated organizations, or those of the publisher, the editors and the reviewers. Any product that may be evaluated in this article, or claim that may be made by its manufacturer, is not guaranteed or endorsed by the publisher.
