# Supplementary material for: Soluble Urokinase Plasminogen Activator Receptor (suPAR) as a Biomarker of Systemic Chronic Inflammation
Source: Front Immunol. 2021 Dec 2;12:780641. doi: 10.3389/fimmu.2021.780641 (PMC8674945; doi:10.3389/fimmu.2021.780641)
Supplement: Supplementary file 1 [file Table_1.docx]

| **Appendix I - Correlation between suPAR and other inflammatory biomarkers** | | | |
| --- | --- | --- | --- |
| **Biomarker, setting** | **Correlation** | **P value** | **Reference** |
| **CRP** |  |  |  |
| General population (n=1,068) | *r* 0.23 | <0.001 | Botha (2014)^1^ |
| General population, survivors (n=1,217) | Positive correlation | <0.001 | Botha (2015)^2^ |
| General population, non-survivors (n=208) | *r* 0.30 | <0.001 | Botha (2015)^2^ |
| General population (n=1,179) | *r* 0.18 | <0.0001 | Diederichsen (2018)^3^ |
| General population (n=2,602) | *ρ* 0.30 | <0.0001 | Eugen-Olsen (2010)^4^ |
| General population (n=830) | *r* 0.15 | <0.001 | Rasmussen (2019)^5^ |
| General population (n=168) | *ρ* 0.18 (0.02–0.33) | <0.001 | Schenk (2019)^6^ |
| Healthy infants (n=18) | No correlation | 0.48 | Siahanidou (2014)^7^ |
| Healthy volunteers (n=94) | *τ* 0.16 | <0.001 | Eugen-Olsen (2016)^8^ |
| Acute Puumala hantavirus (n=97) | *r* 0.298 | 0.003 | Outinen (2013)^9^ |
| AECOPD (n=43) | *r* 0.33 | 0.03 | Gumus (2015)^10^ |
| AMU patients (n=540) | *r* 0.28 | <0.0001 | Haupt (2012)^11^ |
| AMU patients (n=4,343) | 0.36 Kendall’s tau-b | <0.0001 | Rasmussen (2016)^12^ |
| Cirrhosis, decompensated (n=162) | *r* 0.26 | 0.001 | Zimmermann (2013)^13^ |
| Chronic hepatitis C virus (n=38) | *r* 0.36 | 0.027 | Sjöwall (2015)^14^ |
| Chronic liver disease (n=159) | *r* 0.347 | <0.001 | Zimmermann (2012)^15^ |
| COPD patients (n=24) | *r* 0.53 | <0.01 | Böcskei (2019)^16^ |
| Crimean-Congo hemorrhagic fever (n=100) | *r* 0.519 | <0.001 | Yilmaz (2011)^17^ |
| Crohn’s disease (n=22) (ΔCRP, ΔsuPAR) | *r* 0.33 | <0.05 | Lönnkvist (2011)^18^ |
| CVD, carotid plaques (n=162) | *r* 0.268 | 0.001 | Edsfeldt (2012)^19^ |
| CVD, STEMI (n=296) | *r* 0.39 | <0.0001 | Lyngbæk (2012)^20^ |
| Diabetes (Type 1) + controls (n=667 + 51) | *r* 0.24 | <0.001 | Theilade (2015)^21^ |
| DOC patients (n=179) | 0.25 Kendall’s tau-b | <0.0001 | Rasmussen (2017)^22^ |
| ICU patients (n=258) | *r* 0.254 | <0.001 | Donadello (2014)^23^ |
| ICU patients (n=273) | *r* 0.41 | <0.001 | Koch (2011)^24^ |
| Multiple myeloma (n=46) | *r* 0.2257 | n.s. | Rigolin (2003)^25^ |
| NAFLD (n=82) | No correlation | n.s. | Sjöwall (2015)^14^ |
| Neonatal bacterial infections (n=19) | *r* 0.52 | 0.02 | Siahanidou (2014)^7^ |
| Neonatal viral infections (n=28) | No correlation | 0.45 | Siahanidou (2014)^7^ |
| Pediatric inflammatory bowel disease  (n=37) | No correlation | n.s. | Kolho (2012)^26^ |
| Pediatric pneumonia (n=227) | *r* 0.15 | <0.05 | Wrotek (2015)^27^ |
| Pediatric urinary tract infection (n=42) | *r* 0.492 | <0.01 | Wittenhagen (2011)^28^ |
| Psychiatric patients (MDD+SA, n=71) | *rho* 0.52 | <.001 | Ventorp (2015)^29^ |
| Psychiatric patients (MDD+controls, n=34) | No correlation | n.s. | Ventorp (2017)^30^ |
| Schizophrenia + controls (n=174 + 158) | *r* 0.28 | Significant | Bigseth (2021)^31^ |
| End-stage renal disease, hemodialysis  (n=64) | *r* 0.23 | 0.15 | Wlazeł (2018)^32^ |
| Rheumatoid arthritis (n=51) | *r* 0.44 | <0.01 | Slot (1999)^33^ |
| Rheumatoid arthritis (n=61) | *r* 0.32 | 0.02 | Toldi (2013)^34^ |
| SLE (n=198) | *β* 0.36 | <0.0005 | Enocsson (2013)^35^ |
| Sepsis (n=40) | No correlation | n.s. | Khater (2016)^36^ |
| Sepsis (n=27) | *r* 0.15 | 0.53 | Gustafsson (2012)^37^ |
| **Endothelin-1** |  |  |  |
| COPD patients (n=24) | *r* 0.54 | <0.01 | Böcskei (2019)^16^ |
| **Erythrocyte sedimentation rate (ESR)** |  |  |  |
| AECOPD (n=43) | *r* 0.22 | 0.16 | Gumus (2015)^10^ |
| DOC patients (n=191) | 0.27 Kendall’s tau-b | <0.0001 | Rasmussen (2017)^22^ |
| Paediatric inflammatory bowel disease   (n=37) | *r* 0.13 | n.s. | Kolho (2012)^26^ |
| Rheumatoid arthritis (n=51) | *r* 0.35 | <0.05 | Slot (1999)^33^ |
| Rheumatoid arthritis (n=61) | *r* 0.30 | 0.05 | Toldi (2013)^34^ |
| SLE (n=198) | *β* 0.24 | 0.002 | Enocsson (2013)^35^ |
| **Fibrinogen** |  |  |  |
| General population (n=819) | *r* 0.19 | <0.001 | Rasmussen (2019)^5^ |
| AECOPD (n=45) | *r* 0.715 | <0.001 | AboEl-Magd (2018)^38^ |
| AECOPD (n=43) | *r* 0.59 | <0.001 | Gumus (2015)^10^ |
| **IL-1β** |  |  |  |
| SLE (n=198) | *β* 0.37 | <0.0005 | Enocsson (2013)^35^ |
| **IL-6** |  |  |  |
| General population, survivors (n=1,217) | Not reported | <0.001 | Botha (2015)^2^ |
| General population, non-survivors (n=208) | *r* 0.39 | <0.001 | Botha (2015)^2^ |
| General population (n=790) | *r* 0.08 | 0.02 | Rasmussen (2019)^5^ |
| Healthy subjects (n=39) | *ρ* -0.01 | 0.97 | Ostrowski (2005)^39^ |
| Acute Puumala hantavirus (n=97) | *r* 0.621 | <0.001 | Outinen (2013)^9^ |
| Chronic liver disease (n=159) | *r* 0.650 | <0.001 | Zimmermann (2012)^15^ |
| Cirrhosis, decompensated (n=162) | *r* -0.01 | 0.93 | Zimmermann (2013)^13^ |
| COPD patients (n=24) | *r* 0.40 | 0.051 | Böcskei (2019)^16^ |
| HIV (n=36) | 0.26 | 0.13 | Andersen (2008)^40^ |
| SLE (n=198) | No correlation | n.s. | Enocsson (2013)^35^ |
| Pregnancy (n=60) | No correlation | n.s. | Desdicioglu (2017)^41^ |
| Sepsis patients (n=27) and controls (n=22) | *r* 0.04 | 0.84 | Gustafsson (2012)^37^ |
| SIRS (n=50) | *r* 0.395 | 0.004 | Yu (2011)^42^ |
| **IL-8 (CXCL8)** |  |  |  |
| Chronic liver disease (n=159) | *r* 0.412 | <0.001 | Zimmermann (2012)^15^ |
| **IL-10** |  |  |  |
| Chronic liver disease (n=159) | *r* 0.195 | 0.027 | Zimmermann (2012)^15^ |
| Cirrhosis, decompensated (n=162) | *r* 0.22 | 0.006 | Zimmermann (2013)^13^ |
| SLE (n=198) | *β* 0.31 | <0.0005 | Enocsson (2013)^35^ |
| Sepsis patients (n=27) and controls (n=22) | *r* 0.02 | 0.93 | Gustafsson (2012)^37^ |
| **IL-18** |  |  |  |
| Haemodialysis (n=84) | *r* 0.38 | <0.001 | Almroth (2016)^43^ |
| Healthy controls (n=61) | *r* 0.44 | 0.010 | Almroth (2016)^43^ |
|  |  |  |  |
| **LPS-binding protein** |  |  |  |
| Cirrhosis, decompensated (n=162) | *r* 0.14 | 0.07 | Zimmermann (2013)^13^ |
| **MCP-1 (CCL2)** |  |  |  |
| Chronic liver disease (n=159) | *r* 0.174 | 0.034 | Zimmermann (2012)^15^ |
| **Pentraxin-3** |  |  |  |
| Acute Puumala hantavirus (n=97) | *r* 0.425 | 0.005 | Outinen (2013)^9^ |
| **Procalcitonin** |  |  |  |
| Severe acute pancreatitis (n=50) | *r* 0.233 | 0.104 | Long (2019)^44^ |
| ICU patients (n=273) | *r* 0.468 | <0.001 | Koch (2011)^24^ |
| Pediatric pneumonia (n=227) | *r* 0.29 | <0.05 | Wrotek (2015)^27^ |
| Pediatric urinary tract infection (n=14) | *r* 0.300 | n.s. | Wittenhagen (2011)^28^ |
| Sepsis patients (n=27) and controls (n=22) | *r* 0.006 | 0.98 | Gustafsson (2012)^37^ |
| SIRS (n=29) and sepsis (n=82) | *r* 0.326 | <0.001 | Zeng (2016)^45^ |
| **TNF-α** |  |  |  |
| Chronic liver disease (n=159) | *r* 0.534 | <0.001 | Zimmermann (2012)^15^ |
| Cirrhosis, decompensated (n=162) | *r* 0.03 | 0.67 | Zimmermann (2013)^13^ |
| Haemodialysis (n=84) | *r* 0.29 | 0.007 | Almroth (2016)^43^ |
| Healthy controls (n=61) | *r* 0.24 | 0.097 | Almroth (2016)^43^ |
| Healthy subjects (n=39) | *ρ* 0.25 | 0.154 | Ostrowski (2005)^39^ |
| HIV (n=36) | *r* 0.54 | <0.001 | Andersen (2008)^40^ |
| ICU patients (n=273) | *r* 0.57 | <0.001 | Koch (2011)^24^ |
| SIRS (n=50) | *r* 0.606 | <0.001 | Yu (2011)^42^ |
| SLE (n=198) | *β* 0.34 | <0.0005 | Enocsson (2013)^35^ |
| Trauma patients (n=51) | *r* 0.43 | <0.01 | Timmermans (2015)^46^ |
| **White blood cell (WBC) count** |  |  |  |
| Acute Puumala hantavirus (n=97) | *r* 0.475 | <0.001 | Outinen (2013)^9^ |
| AECOPD (n=43) | *r* 0.09 | 0.55 | Gumus (2015)^10^ |
| AMU patients (n=539) | *r* 0.16 | <0.0001 | Haupt (2012)^11^ |
| Chronic liver disease (n=159) | *r* 0.177 | 0.028 | Zimmermann (2012)^15^ |
| Cirrhosis, decompensated (n=162) | *r* 0.32 | <0.001 | Zimmermann (2013)^13^ |
| Crimean-Congo haemorrhagic fever   (n=100) | *r* 0.547 | <0.001 | Yilmaz (2011)^17^ |
| General population (n=827) | *r* 0.22 | <0.001 | Rasmussen (2019)^5^ |
| Healthy volunteers (n=94) | 0.36 Kendall’s tau-b | <0.001 | Eugen-Olsen (2016)^8^ |
| Liver disease (n=159) | *r* 0.177 | 0.028 | Zimmermann (2012)^15^ |
| Neonatal infections + controls (n=47 + 18) | *r* 0.26 | 0.03 | Siahanidou (2014)^7^ |
| Pediatric malaria (n=478) | *r* 0.14 | 0.005 | Ostrowski (2005)^47^ |
| Pediatric pneumonia (n=227) | - | n.s. | Wrotek (2015)^27^ |
| **White blood cell types:** |  |  |  |
| **Basophil count** |  |  |  |
| General population (n=827) | *r* 0.04 | 0.29 | Rasmussen (2019)^5^ |
| Healthy volunteers (n=94) | 0.07 Kendall’s tau-b | n.s. | Eugen-Olsen (2016)^8^ |
| **Eosinophil count** |  |  |  |
| General population (n=827) | *r* 0.05 | 0.17 | Rasmussen (2019)^5^ |
| Healthy volunteers (n=94) | 0.19 Kendall’s tau-b | 0.009 | Eugen-Olsen (2016)^8^ |
| **Lymphocyte count** |  |  |  |
| General population (n=827) | *r* 0.18 | <0.001 | Rasmussen (2019)^5^ |
| Healthy volunteers (n=94) | 0.09 Kendall’s tau-b | n.s. | Eugen-Olsen (2016)^8^ |
| Pediatric malaria (n=478) | *r* 0.07 | 0.171 | Ostrowski (2005)^47^ |
| Pediatric pneumonia (n=227) | - | n.s. | Wrotek (2015)^27^ |
| **Monocyte count** |  |  |  |
| General population (n=827) | *r* 0.18 | <0.001 | Rasmussen (2019)^5^ |
| Healthy volunteers (n=94) | 0.19 Kendall’s tau-b | 0.009 | Eugen-Olsen (2016)^8^ |
| **Neutrophil count** |  |  |  |
| General population (n=827) | *r* 0.16 | <0.001 | Rasmussen (2019)^5^ |
| Healthy volunteers (n=94) | 0.38 Kendall’s tau-b | <0.001 | Eugen-Olsen (2016)^8^ |
| Chronic liver disease (n=159) | *r* 0.311 | <0.001 | Zimmermann (2012)^15^ |
| Paediatric pneumonia (n=227) | No correlation | n.s. | Wrotek (2015)^27^ |
| **Note:** This overview is not exhaustive.  Abbreviations: AECOPD, acute exacerbations of chronic obstructive pulmonary disease; AMU, Acute Medical Unit; COPD, chronic obstructive pulmonary disease; CRP, C-reactive protein; CVD, cardiovascular disease; DOC, Diagnostic Outpatient Clinic; HIV, human immunodeficiency virus; ICU, intensive care unit; IL, interleukin; LPS, lipopolysaccharide; MCP-1, monocyte chemoattractant protein-1; MDD, major depressive disorder; n.s., not significant; NAFLD, non-alcoholic fatty liver disease; SA, suicide attempt; SIRS, systemic inflammatory response syndrome; SLE, systemic lupus erythematosus; STEMI, ST-segment elevation myocardial infarction; suPAR, soluble urokinase plasminogen activator receptor; TNF, tumor necrosis factor. | | | |

**References**

1. Botha S, Fourie CMT, Schutte R, Kruger A, Schutte AE. Associations of suPAR with lifestyle and cardiometabolic risk factors. *Eur J Clin Invest*. 2014;44(7):619-626. doi:10.1111/eci.12278

2. Botha S, Fourie CM, Schutte R, Eugen-Olsen J, Pretorius R, Schutte AE. Soluble urokinase plasminogen activator receptor as a prognostic marker of all-cause and cardiovascular mortality in a black population. *Int J Cardiol*. 2015;184:631-636. doi:10.1016/j.ijcard.2015.03.041

3. Diederichsen MZ, Diederichsen SZ, Mickley H, et al. Prognostic value of suPAR and hs-CRP on cardiovascular disease. *Atherosclerosis*. 2018;271:245-251. doi:10.1016/j.atherosclerosis.2018.01.029

4. Eugen-Olsen J, Andersen O, Linneberg A, et al. Circulating soluble urokinase plasminogen activator receptor predicts cancer, cardiovascular disease, diabetes and mortality in the general population. *J Intern Med*. 2010;268(3):296-308. doi:10.1111/j.1365-2796.2010.02252.x

5. Rasmussen LJH, Moffitt TE, Eugen-Olsen J, et al. Cumulative childhood risk is associated with a new measure of chronic inflammation in adulthood. *J Child Psychol Psychiatry*. 2019;60(2):199-208. doi:10.1111/jcpp.12928

6. Schenk M, Eichelmann F, Schulze MB, et al. Reproducibility of novel immune-inflammatory biomarkers over 4 months: an analysis with repeated measures design. *Biomark Med*. 2019;13(8):639-648. doi:10.2217/bmm-2018-0351

7. Siahanidou T, Margeli A, Tsirogianni C, et al. Clinical value of plasma soluble Urokinase-type plasminogen activator receptor levels in term neonates with infection or sepsis: A prospective study. *Mediators Inflamm*. 2014;2014. doi:10.1155/2014/375702

8. Eugen-Olsen J, Ladelund S, Sørensen LT. Plasma suPAR is lowered by smoking cessation: A randomized controlled study. *Eur J Clin Invest*. 2016;46(4):305-311. doi:10.1111/eci.12593

9. Outinen TK, Tervo L, Mäkelä S, et al. Plasma Levels of Soluble Urokinase-Type Plasminogen Activator Receptor Associate with the Clinical Severity of Acute Puumala Hantavirus Infection. *PLoS One*. 2013;8(8):e71335. doi:10.1371/journal.pone.0071335

10. Gumus A, Altintas N, Cinarka H, et al. Soluble urokinase-type plasminogen activator receptor is a novel biomarker predicting acute exacerbation in COPD. *Int J Chron Obstruct Pulmon Dis*. 2015;10:357-365. doi:10.2147/COPD.S77654

11. Haupt TH, Petersen J, Ellekilde G, et al. Plasma suPAR levels are associated with mortality, admission time, and Charlson Comorbidity Index in the acutely admitted medical patient: a prospective observational study. *Crit Care*. 2012;16(4):R130. doi:10.1186/cc11434

12. Rasmussen LJH, Ladelund S, Haupt TH, et al. Soluble urokinase plasminogen activator receptor (suPAR) in acute care: A strong marker of disease presence and severity, readmission and mortality. A retrospective cohort study. *Emerg Med J*. 2016;33(11):769-775. doi:10.1136/emermed-2015-205444

13. Zimmermann HW, Reuken PA, Koch A, et al. Soluble urokinase plasminogen activator receptor is compartmentally regulated in decompensated cirrhosis and indicates immune activation and short-term mortality. *J Intern Med*. 2013;274(1):86-100. doi:10.1111/joim.12054

14. Sjöwall C, Martinsson K, Cardell K, Ekstedt M, Kechagias S. Soluble urokinase plasminogen activator receptor levels are associated with severity of fibrosis in nonalcoholic fatty liver disease. *Transl Res*. 2015;165(6):658-666. doi:10.1016/j.trsl.2014.09.007

15. Zimmermann HW, Koch A, Seidler S, Trautwein C, Tacke F. Circulating soluble urokinase plasminogen activator is elevated in patients with chronic liver disease, discriminates stage and aetiology of cirrhosis and predicts prognosis. *Liver Int*. 2012;32(3):500-509. doi:10.1111/j.1478-3231.2011.02665.x

16. Böcskei RM, Benczúr B, Losonczy G, et al. Soluble Urokinase-Type Plasminogen Activator Receptor and Arterial Stiffness in Patients with COPD. *Lung*. 2019;197(2):189-197. doi:10.1007/s00408-019-00211-w

17. Yilmaz G, Mentese A, Kaya S, Uzun A, Karahan SC, Koksal I. The diagnostic and prognostic significance of soluble urokinase plasminogen activator receptor in Crimean-Congo hemorrhagic fever. *J Clin Virol*. 2011;50(3):209-211. doi:10.1016/j.jcv.2010.11.014

18. Lönnkvist MH, Theodorsson E, Holst M, Ljung T, Hellström PM. Blood chemistry markers for evaluation of inflammatory activity in Crohn’s disease during infliximab therapy. *Scand J Gastroenterol*. 2011;46(4):420-427. doi:10.3109/00365521.2010.539253

19. Edsfeldt A, Nitulescu M, Grufman H, et al. Soluble urokinase plasminogen activator receptor is associated with inflammation in the vulnerable human atherosclerotic plaque. *Stroke*. 2012;43(12):3305-3312. doi:10.1161/STROKEAHA.112.664094

20. Lyngbæk S, Marott JL, Møller D V, et al. Usefulness of soluble urokinase plasminogen activator receptor to predict repeat myocardial infarction and mortality in patients with ST-segment elevation myocardial infarction undergoing primary percutaneous intervention. *Am J Cardiol*. 2012;110(12):1756-1763. doi:10.1016/j.amjcard.2012.08.008

21. Theilade S, Lyngbaek S, Hansen TW, et al. Soluble urokinase plasminogen activator receptor levels are elevated and associated with complications in patients with type 1 diabetes. *J Intern Med*. 2015;277(3):362-371. doi:10.1111/joim.12269

22. Rasmussen LJH, Schultz M, Gaardsting A, et al. Inflammatory biomarkers and cancer: CRP and suPAR as markers of incident cancer in patients with serious nonspecific symptoms and signs of cancer. *Int J Cancer*. 2017;141(1):191-199. doi:10.1002/ijc.30732

23. Donadello K, Scolletta S, Taccone FS, et al. Soluble urokinase-type plasminogen activator receptor as a prognostic biomarker in critically ill patients. *J Crit Care*. 2014;29(1):144-149. doi:10.1016/j.jcrc.2013.08.005

24. Koch A, Voigt S, Kruschinski C, et al. Circulating soluble urokinase plasminogen activator receptor is stably elevated during the first week of treatment in the intensive care unit and predicts mortality in critically ill patients. *Crit Care*. 2011;15(1):R63. doi:10.1186/cc10037

25. Rigolin GM, Tieghi A, Ciccone M, et al. Soluble urokinase-type plasminogen activator receptor (suPAR) as an independent factor predicting worse prognosis and extra-bone marrow involvement in multiple myeloma patients. *Br J Haematol*. 2003;120(6):953-959. doi:10.1046/j.1365-2141.2003.04176.x

26. Kolho K-L, Valtonen E, Rintamäki H, Savilahti E. Soluble urokinase plasminogen activator receptor suPAR as a marker for inflammation in pediatric inflammatory bowel disease. *Scand J Gastroenterol*. 2012;47(8-9):951-955. doi:10.3109/00365521.2012.699549

27. Wrotek A, Jackowska T, Pawlik K. Soluble urokinase plasminogen activator receptor: an indicator of pneumonia severity in children. *Adv Exp Med Biol*. 2015;835:1-7. doi:10.1007/5584_2014_40

28. Wittenhagen P, Andersen JB, Hansen A, et al. Plasma soluble urokinase plasminogen activator receptor in children with urinary tract infection. *Biomark Insights*. 2011;6:79-82. doi:10.4137/BMI.S6876

29. Ventorp F, Gustafsson A, Träskman-Bendz L, Westrin Å, Ljunggren L. Increased soluble urokinase-type plasminogen activator receptor (suPAR) levels in plasma of suicide attempters. *PLoS One*. 2015;10(10):e0140052. doi:10.1371/journal.pone.0140052

30. Gustafsson A, Ventorp F, Wisén AGM, Ohlsson L, Ljunggren L, Westrin Å. Effects of acute exercise on circulating soluble form of the urokinase receptor in patients with major depressive disorder. *Biomark Insights*. 2017;12. doi:10.1177/1177271917704193

31. Bigseth TT, Fredriksen M, Egeland J, et al. Elevated levels of soluble urokinase plasminogen activator receptor as a low-grade inflammation marker in schizophrenia: A case-control study. *Schizophr Res*. 2021;228:190-192. doi:10.1016/j.schres.2020.11.051

32. Wlazeł RN, Szadkowska I, Bartnicki P, Rośniak-Bąk K, Rysz J. Clinical and prognostic usefulness of soluble urokinase plasminogen activator receptor in hemodialysis patients. *Int Urol Nephrol*. 2018;50(2):339-345. doi:10.1007/s11255-017-1778-5

33. Slot O, Brünner N, Locht H, Oxholm P, Stephens RW. Soluble urokinase plasminogen activator receptor in plasma of patients with inflammatory rheumatic disorders: Increased concentrations in rheumatoid arthritis. *Ann Rheum Dis*. 1999;58(8):488-492. doi:10.1136/ard.58.8.488

34. Toldi G, Bekő G, Kádár G, et al. Soluble urokinase plasminogen activator receptor (suPAR) in the assessment of inflammatory activity of rheumatoid arthritis patients in remission. *Clin Chem Lab Med*. 2013;51(2):327-332. doi:10.1515/cclm-2012-0221

35. Enocsson H, Wetterö J, Skogh T, Sjöwall C. Soluble urokinase plasminogen activator receptor levels reflect organ damage in systemic lupus erythematosus. *Transl Res*. 2013;162(5):287-296. doi:10.1016/j.trsl.2013.07.003

36. Khater WS, Salah-Eldeen NN, Khater MS, Saleh AN. Role of suPAR and lactic acid in diagnosing sepsis and predicting mortality in elderly patients. *Eur J Microbiol Immunol*. 2016;6(3):178-185. doi:10.1556/1886.2016.00011

37. Gustafsson A, Ljunggren L, Bodelsson M, Berkestedt I. The prognostic value of suPAR compared to other inflammatory markers in patients with severe sepsis. *Biomark Insights*. 2012;7:39-44. doi:10.4137/BMI.S9460

38. AboEl-Magd GH, Mabrouk MM. Soluble urokinase-type plasminogen activator receptor as a measure of treatment response in acute exacerbation of COPD. *J Bras Pneumol*. 2018;44(1):36-41. doi:10.1590/S1806-37562017000000151

39. Ostrowski SR, Plomgaard P, Fischer CP, et al. Interleukin-6 infusion during human endotoxaemia inhibits in vitro release of the urokinase receptor from peripheral blood mononuclear cells. *Scand J Immunol*. 2005;61(2):197-206. doi:10.1111/j.0300-9475.2005.01547.x

40. Andersen O, Eugen-Olsen J, Kofoed K, Iversen J, Haugaard SB. Soluble Urokinase Plasminogen Activator Receptor is a Marker of Dysmetabolism in HIV-Infected Patients Receiving Highly Active Antiretroviral Therapy. *J Med Virol*. 2008;80(2):209-216. doi:10.1002/jmv.21114

41. Desdicioglu R, Yildirim M, Kocaoglu G, et al. Soluble urokinase-type plasminogen activator receptor (suPAR) and interleukin-6 levels in hyperemesis gravidarum. *J Chinese Med Assoc*. 2018;81(9):825-829. doi:10.1016/j.jcma.2017.08.013

42. Yu L, Long D, Wu X-L, Yang J, Yang Y, Feng G. Prognostic significance of urokinase-type plasminogen activator and its receptor in patients with systemic inflammatory response syndrome. *World J Emerg Med*. 2011;2(3):185. doi:10.5847/wjem.j.1920-8642.2011.03.005

43. Almroth G, Lönn J, Uhlin F, Brudin L, Andersson B, Hahn-Zoric M. Sclerostin, TNF-alpha and Interleukin-18 Correlate and are Together with Klotho Related to Other Growth Factors and Cytokines in Haemodialysis Patients. *Scand J Immunol*. 2016;83(1):58-63. doi:10.1111/sji.12392

44. Long D, Wang Y, Wang H, Wu X, Yu L. Correlation of Serum and Ascitic Fluid Soluble Form Urokinase Plasminogen Activator Receptor Levels With Patient Complications, Disease Severity, Inflammatory Markers, and Prognosis in Patients With Severe Acute Pancreatitis. *Pancreas*. 2019;48(3):335-342. doi:10.1097/MPA.0000000000001247

45. Zeng M, Chang M, Zheng H, et al. Clinical value of soluble urokinase-type plasminogen activator receptor in the diagnosis, prognosis, and therapeutic guidance of sepsis. *Am J Emerg Med*. 2016;34(3):375-380. doi:10.1016/j.ajem.2015.11.004

46. Timmermans K, Vaneker M, Scheffer GJ, et al. Soluble urokinase-type plasminogen activator levels are related to plasma cytokine levels but have low predictive value for mortality in trauma patients. *J Crit Care*. 2015;30(3):476-480. doi:10.1016/j.jcrc.2015.01.006

47. Ostrowski SR, Ullum H, Goka BQ, et al. Plasma Concentrations of Soluble Urokinase‐Type Plasminogen Activator Receptor Are Increased in Patients with Malaria and Are Associated with a Poor Clinical or a Fatal Outcome. *J Infect Dis*. 2005;191(8):1331-1341. doi:10.1086/428854
